# Synthesis, Structure and In Vitro Anti-Trypanosomal Activity of Non-Toxic Arylpyrrole-Based Chalcone Derivatives

**DOI:** 10.3390/molecules25071668

**Published:** 2020-04-04

**Authors:** Ayanda I. Zulu, Ogunyemi O. Oderinlo, Cuan Kruger, Michelle Isaacs, Heinrich C. Hoppe, Vincent J. Smith, Clinton G. L. Veale, Setshaba D. Khanye

**Affiliations:** 1 Department of Chemistry, Faculty of Science, Rhodes University, Grahamstown 6140, South Africa; azulu50@gmail.com (A.I.Z.); oderinloyemi@yahoo.com (O.O.O.); g13k8200@campus.ru.ac.za (C.K.); v.smith@ru.ac.za (V.J.S.); 2 Centre for Chemico and Biomedicinal Research, Rhodes University, Grahamstown 6140, South Africa; m.isaacs@ru.ac.za (M.I.); h.hoppe@ru.ac.za (H.C.H.); 3 Department of Biochemistry and Microbiology, Faculty of Science, Rhodes University, Grahamstown 6140, South Africa; 4 School of Chemistry and Physics, Pietermaritzburg Campus, University of KwaZulu-Natal, Private Bag X01, Scottsville 3209, South Africa; VealeC@ukzn.ac.za; 5 Division of Pharmaceutical Chemistry, Faculty of Pharmacy, Rhodes University, Grahamstown 6140, South Africa

**Keywords:** arylpyrrole, chalcones, trypanosomiasis, *Trypanosoma brucei*, molecular hybridization

## Abstract

With an intention of identifying chalcone derivatives exhibiting anti-protozoal activity, a cohort of relatively unexplored arylpyrrole-based chalcone derivatives were synthesized in moderate to good yields. The resultant compounds were evaluated in vitro for their potential activity against a cultured *Trypanosoma brucei brucei* 427 strain. Several compounds displayed mostly modest in vitro anti-trypanosomal activity with compounds **10e** and **10h** emerging as active candidates with IC_50_ values of 4.09 and 5.11 µM, respectively. More importantly, a concomitant assessment of their activity against a human cervix adenocarcinoma (HeLa) cell line revealed that these compounds are non-toxic.

## 1. Introduction

Infectious diseases caused by kinetoplastid parasites are a serious health threat worldwide. Among these is human African trypanosomiasis (HAT), commonly referred to as sleeping sickness [1,2]. HAT has also been prioritized by the World Health Organization (WHO) in the list of neglected tropical diseases (NTDs) in 149 endemic countries [3,4,5]. A significantly large population mainly in poor rural locations of sub-Saharan Africa remains at risk of contracting the disease [5,6]. The causative agents of HAT are protozoan trypanosomes of the genus *Trypanosoma*: *T. brucei rhodesiense* and *T. brucei gambiense* [7,8]. These are transmitted by the insect vector tsetse fly of the *Glossina* species, which injects metacyclic trypomastigotes into the human host [9]. *T. b. gambiense*, which is common to west and central Africa, is responsible for >98% of reported cases of HAT in the continent of Africa [10], whilst *T. b. rhodesiense* is found in eastern and southern Africa and accounts for less than 2% of reported cases.

**Figure 1:**
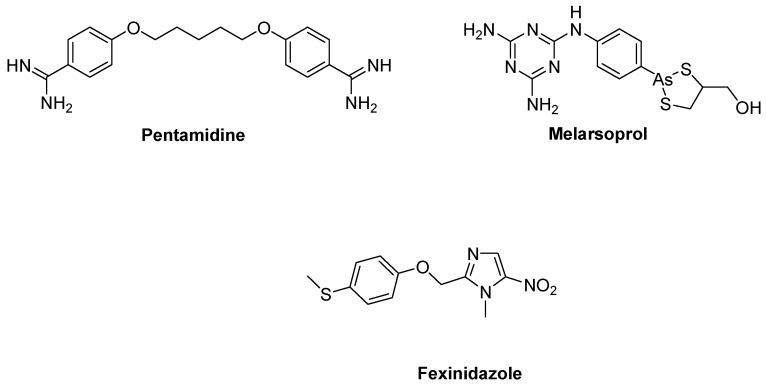
Examples of some drugs used for the treatment of human African sleeping sickness.

Despite their discovery almost a century ago, the most successful agents in clinical use for the treatment of HAT are pentamidine, melarsoprol (Figure 1), suramin, and eflornithine [11]. However, these drugs suffer from some limitations, including poor oral bioavailability and complex and time-consuming intravenous (IV) administration, which is costly and requires highly skilled health practitioners and facilities that are limited in poverty-stricken rural areas [12,13], and thus impact the implementation of treatment [14]. Additionally, currently available anti-trypanosomal drugs have serious side effects, such as nephrotoxicity, hypertension, anemia, and neuropathy [15,16]. In the absence of effective vaccines to prevent infections inflicted by HAT, treatment efforts are solely reliant on chemotherapeutic intervention [3,17]. Treatment options are implemented such that for stage 1 of the disease pentamidine and suramin are utilized, despite their limited penetration of the central nervous system (CNS) [18]. For stage 2 of the disease, melarsoprol and eflornithine are the preferred treatment options, however, these drugs suffer from issues relating to toxicity and trypanosomal resistance [19]. Considering the urgent need to develop therapeutic alternatives that target both stages of trypanosomiasis, a partnership between Sanofi and Drug for Neglected Diseases initiative (DND*i*) has led to the approval of fexinidazole (Figure 1) as an alternative therapeutic armament for tackling both stage 1 and 2 HAT [1,20]. However, the ever-growing concern regarding resistance against clinically approved drugs, and the potential for an epidemic outbreak, has resulted in several campaigns focused on identifying new compounds with novel modes of action to prevent and control trypanosomal infections [21,22,23].

**Figure 2:**
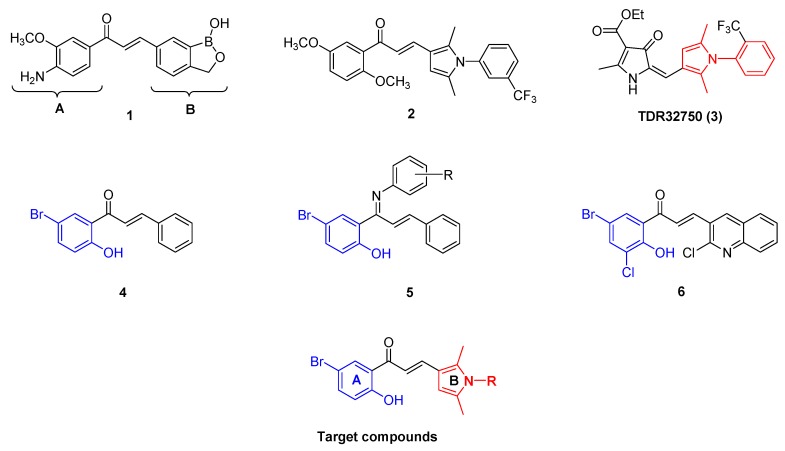
Examples of some bioactive chalcone derivatives, arylpyrroles, and target compounds.

Chalcones are open-chain flavonoids, which feature a characteristic three carbon α,β-unsaturated carbonyl system [24,25]. More importantly, chalcones are appealing as key synthetic intermediates and frameworks in the design of therapeutic tools for the treatment of various ailments including antimicrobial, antibacterial, antimalarial, anticancer, anti-inflammatory, anti-HIV, anti-Alzheimer’s, etc. [24,25,26,27,28,29,30,31,32]. The structural modification of chalcones where the B-ring (Figure 2) is substituted with other bioactive fragments or units is a contemporary strategy that has been extensively utilized by various research groups involved in designing bioactive compounds for the treatment of different diseases [33]. A typical example is that of Zhou and co-workers, who prepared a series of novel chalcones by incorporating the benzoxaborole motif to form hybrid molecules (Figure 2) showing excellent in vivo activity in a murine model of *T. b. brucei*, with compound **1** emerging as the most active compound, with an IC_50_ value of 0.010 µg/mL [34].

In an effort to identify compounds with desirable potency against methicillin-resistant *Staphylococcus aureus* (MRSA), Osório and co-workers reported a focused library of chalcone derivatives, including compound **2**, bearing an *N*-arylpyrrole moiety [35]. Thus far, compound **2** remains unexplored for antiprotozoal activity, with no examples of analogues appearing in the literature. Importantly, in our context, several studies have shown that compounds containing the arylpyrrole framework display a broad spectrum of biological properties, including antiparasitic activities, as illustrated by the anti-malarial lead compound **3** [36]. Furthermore, numerous chalcone derivatives, including compounds **4** and **5**, have been reported as possessing anti-bacterial and anti-tuberculosis activity [37]. In a separate study, Dave et al. explored chalcone derivatives in which the B-ring was substituted for a quinoline nucleus, for their potential as antiplasmodial agents. In that study, compound **6** displayed favorable anti-plasmodial potency, with the 5-bromo-2-hydroxy substitution pattern on ring A (portion in blue) appearing to be a crucial element of the pharmacophore [38].

Molecular hybridization, which combines two or more dissimilar pharmacophoric subunits to improve efficacy and other pharmaceutical profiles is becoming an attractive approach to design novel biologically active compounds [39,40,41]. Considering our ongoing research interests to identify and develop new antiparasitic compounds [42,43,44], we employed a molecular hybridization approach to design a target series of chalcone derivatives (Figure 2) featuring A ring with hydroxyl and bromine substituents at position 2 and 5, respectively, and arylpyrrole containing B ring system. Herein, we wish to report the synthesis of a representative series of new and non-toxic arylpyrrole-based chalcone derivatives, along with their in vitro anti-trypanosomal effects against the nagana *T. b. brucei* strain.

## 2. Results and Discussion

### 2.1. Chemical Synthesis

**Scheme 1:**
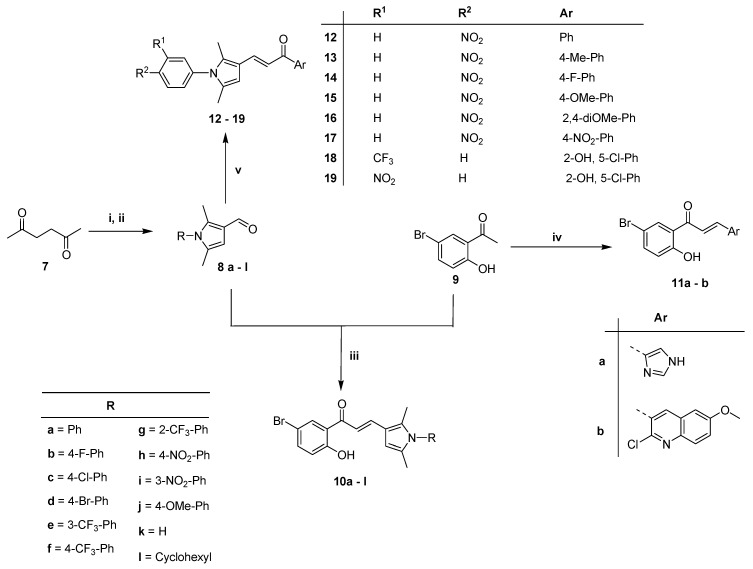
Reagents and conditions: (i) Anilines or cyclohexalamine, solvent free, 160 °C, 16 h; (ii) POCl_3_, DMF, 0 °C–60 °C, 4 h; (iii) 60% NaOH, MeOH, rt, 12 h; (iv) Aldehydes, 60% NaOH, MeOH, rt, 12 h.

Reaction of commercially available 2,5-hexadione **7** with a variety of anilines under the standard Paal-Knorr condensation reaction conditions (Scheme 1) afforded starting arylpyrrole intermediates (see Supplementary Information) with yields varying between 38%–81% [45]. Subsequently, the Vilsmeier-Haack formylation of resultant arylpyrrole intermediates led to the formation of the desired aldehydes **8a**–**k** in moderate to excellent yields [46]. Subjecting the commercially available 2,5-dimethyl-1*H*-pyrrole under similar Vilsmeier-Haack reaction conditions yielded compound **8l** as a white solid in good yield. Treatment of prepared aldehydes **8a**–**l** with the 2-hydroxy-5-bromoacetophenone **9** under base-catalyzed Claisen-Schmidt condensation conditions, afforded the desired arylpyrrole-chalcone derivatives **10a**–**l**, which were isolated in yields varying between 9%–71%. Similarly, compound **11a** was obtained in a poor yield by treating **8** with 2,5-dimethyl-1*H*-imidazole-4-carbaldehyde, while compound **11b** was synthesized from the 2-chloro-6-methoxyquinoline-3-carbaldehyde at a 39% yield, as previously described in the literature [38]. Further preliminary structure-activity relationship (SAR) investigations were conducted through structural modification of the ring A. The starting commercially available acetophenones were reacted with selected arylpyrrolecarbaldehydes **8e**, **8h**, and **8i** under the same base-catalyzed Claisen-Schmidt reaction conditions (Scheme 1), to form the desired compounds **12**–**19** with yields in the range 5%–89%.

**Figure 3:**
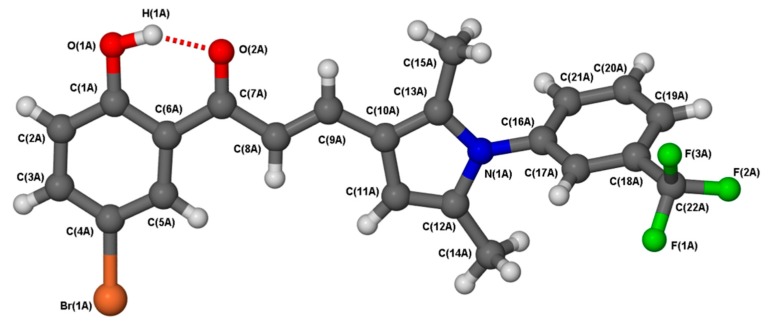
Molecular structure of **10e** depicting the numbering scheme and the intramolecular hydrogen bond (shown here by the red dashed line). The hydrogen atoms on carbons were fixed in geometrically calculated while those bound to oxygen were freely refined. The structure was solved and refined to a final residual factor *R*_1_ = 0.0751. All non-hydrogen atoms were refined anisotropically, with the ellipsoids drawn at the 50% probability level (see Figure S1 in the Supplementary Materials).

All of the synthesized compounds were fully characterized using routine spectroscopic methods (NMR, IR and MS). The coupling constant (*J*) of the α,β-unsaturated protons featured values in the range 12–16 Hz, thus indicating a *trans* configuration [47]. This observation was corroborated by the molecular structure of compound **10e**, determined by single crystal X-ray diffraction analysis. Crystal and experimental data are summarized in Table S1 (Supplementary Information). Compound **10e** crystallizes in the orthorhombic crystal system, in the centrosymmetric space group *Pbca* with eight molecules per unit cell, *Z* = 8. The crystal structure of compound **10e** (Figure 3 and Figure S1) confirmed unequivocally a *trans* configuration about the double bond (C(8A)-C(9A)).

**Figure 4:**
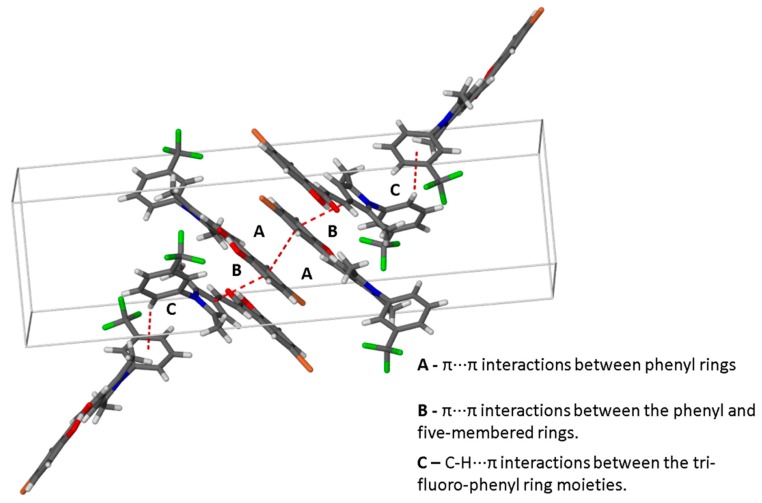
Packing diagram depicting π···π stacking and C-H···π interactions of **10e** that run diagonally across the *bc* plane. The C-F···π interactions have been omitted for clarity.

From the structure of **10e**, bond lengths and bond angles are comparable to chalcone derivatives, which have been reported in the literature [48]. The existence of an α,β-unsaturated ketone is further confirmed by short O(2A)-C(7A) and C(8A)-C(9A) bond lengths of 1.244(7) Å and 1.359(6) Å, respectively; and bond angles O(2A)-C(7A)-C(8A) and C(7A)-C(8A)-C(9A) of 121.2(5)° and 120.8(4)°, respectively. More importantly, the structure exhibits intramolecular hydrogen bonding between the hydroxyl oxygen of ring A and the ketone adjacent to ring A. Introduction of intramolecular hydrogen bond (IMHB) into the bioactive compounds has been reported to have beneficial effects, including biological activity [49]. For example, Attram and co-workers synthesised a novel class of benzimidazoles containing an IMHB structural feature showing in vitro antiplasmodial activity against chloroquine-sensitive (NF54) and multi-drug resistant (K1) strains of the malaria parasite *Plasmodium falciparum* [50]. The aryl moiety on ring B of the chalcone is rotated about the N(1A)-C(16A) bond with corresponding four torsion angles (Figure S2). The observed torsion angles suggest the deviation from planarity of the aryl group attached to the pyrrole framework. The crystal packing (Figure 4) of the molecule is stabilized by π-π stacking interactions between phenyl rings (**A**) and between the phenyl and pyrrole rings (**B**). The centroid-to-centroid distances (**A**,**B**) are approximately 3.927(3) Å and 3.731(2) Å, which further confirms the intermolecular π-π stacking interactions between chalcone molecules in the crystal structure [48]. Additionally, the supramolecular assembly is supported by weak C-H···π and C-F···π interactions between the trifluorophenyl ring moieties of the arylpyrrole (**C**) [C(21A)-H(21A)···π is 2.95 Å and C(22A)-F(2A)···π is 3.32(1) Å].

### 2.2. In Vitro Biological Assessment

A single concentration (20 µM) cytotoxicity evaluation of all compounds was conducted in duplicates using a human cervix adenocarcinoma (HeLa) cell line, with emetine (EMT) included as a reference drug and cell viability relative to untreated cells assessed using resazurin. Compounds **10a–l** possessed negligible cytotoxicity effects against the HeLa cell line, and in general, >80% HeLa cell viability was observed (see Table S2). The anti-trypanosomal activity of prepared compounds against nagana *T. b. brucei* (the subspecies responsible for African trypanosomiasis in cattle) parasite cultures were determined using the *T. b. brucei* 427 strain. Pentamidine (PMD), which is an existing drug for the treatment of trypanosomiasis, was included as a positive control drug. The trypanocidal activity of each compound was initially assessed at a fixed concentration of 20 μM, using resazurin as a read-out of cell viability after compound exposure. All of the compounds which inhibited cell viability below 25% relative to untreated controls at this concentration were taken forward for IC_50_ determination. From Table 1, the structural modification was initially performed on the arylpyrrole moiety (ring B) of the chalcones, while keeping the ring A unchanged. Compound **10a**, with an unsubstituted aromatic ring attached to the arylpyrrole moiety, showed modest activity against *T. b. brucei*, with an IC_50_ value of 11.5 μM. Introduction of the fluorine, highly electronegative atom, at the *para*-position (**10b**) appeared to enhance the activity against *T. b. brucei*; however, the activity diminished when the fluorine was replaced by chlorine (**10c**). Despite **10d** exhibiting similar activity to compound **10a**, inclusion of the bromine substituent (**10d)** slightly restored activity comparable to **10c**. The presence of the trifluoromethyl (CF_3_) functionality at the *para-* position resulted in compound **10e**, whose activity closely mirrored **10b**. The positional isomer of **10e** which features CF_3_ at the *meta-* position (**10f**) displayed reduced activity with an IC_50_ value of 11.4 μM, while the *ortho* substituted analogue **10g** showed moderate activity in line with compounds **10a**, **10d**, and **10f**. Exchanging of CF_3_ with yet another electron withdrawing substituent, NO_2_ at the *para* position (**10h**), also showed improved biological activity with an IC_50_ < 6 μM, while the *meta* isomer (**10i**) possessed modest activity, with an IC_50_ value slightly below 10 μM. Compound **10j**, which features an electron donating methoxy moiety, showed no activity.

Compound **10k**, which lacked an aromatic ring substituent on the pyrrole ring, showed a complete loss of activity against the *T. b. brucei* strain, while compound **10l**, with a saturated cyclohexyl ring instead of either a substituted or non-substituted aromatic ring, showed no improvement in terms of potency, and its IC_50_ value was comparable to that of compounds **10a** and **10f**. Compounds **11a** and **11b**, which featured aromatic replacements of the arylpyrrole moiety, were evaluated for trypanocidal activity. While **11a** showed no activity, compound **11b**, with a quinoline heterocyclic unit, was found to be moderately active against trypanosomes, with an IC_50_ value of 9.1 µM. Having identified compounds **10e** and **10h** as the most active compounds in our initial cohort, we proceeded to explore compounds which maintained the arylpyrrole substituents of either compounds **10e** and **10h**, whilst replacing the 2-hydro-5-bromo-substituted aromatic moiety. These compounds (**12**–**19**) showed moderate inhibitory activity against *T. b. brucei*, with IC_50_ values ranging between 9.3–44.3 µM (Table 1). Despite moderate activity, the data suggest that a combination of the hydroxyl group at position 2 and the bromine at position 5 in the ring A is necessary for activity against the *T. b. brucei* parasite.

## 3. Materials and Methods

### 3.1. General Information

All commercially available reagents were purchased from chemical suppliers, Sigma-Adrich (Pty) Ltd. (Johannesburg, South Africa) and Merck (Pty) Ltd. (Johannesburg, South Africa), and were used without further purification unless stated otherwise. Solvents were distilled before use. The progress of reactions were monitored by thin layer chromatography (TLC) using Merck F_254_ silica gel plates (Merck, Johannesburg, South Africa) supported on aluminium, and were visualized under ultra-violet (UV 254 and 366 nm) light, and, where necessary, stained in an iodine tank. The crude compounds were purified by a silica gel column chromatography using Merck Kieselgel 60 Å: 70–230 (0.068–0.2 mm) silica gel mesh. NMR (^1^H and ^13^C) spectra were recorded on Bruker Biospin 300, 400, or 600 MHz spectrometer. Chemical shifts are reported in ppm and are referenced internally using residual solvent signals (DMSO-*d*_6_ δ_H_ 2.50, δ_C_ 39.5 ppm; CDCl_3_ δ_H_ 7.26, δ_C_ 77.0 ppm). The coupling constants are given in Hertz. High-resolution mass spectrometry (HRMS) was performed on a Waters API Q-TOF Ultima spectrometer (Stellenbosch University, Stellenbosch, South Africa). The IR spectra were recorded on PerkinElmer Spectrum 100 FT-IR Spectrometer (Johannesburg, South Africa) in the mid-IR range (640–4000 cm^−1^). Melting points were measured using a Reichert hot-stage apparatus and are uncorrected. Elemental microanalysis was performed on Elementar Analysensysteme varioMICRO V1.6.2 GmbH analysis system. The calculated values were determined using an online Elemental Composition Calculator from Microanalysis Laboratory, School of Chemical Sciences, University of Illinois at Urbana, USA [51]. Herein, compound **11b** was synthesized from the starting ketone **8** and 2-chloro-6-methoxyquinoline-3-carbaldehyde, according to the method previously described in the literature [38].

### 3.2. General Procedure for the Synthesis of Arylpyrrole-Chalcone Hybrids

A 60% NaOH (10 mL) solution was cooled in an ice bath while stirring, and 5-bromo-2-hydroxyacetophenone (1 eq.) was slowly added and the reaction mixture allowed stirring for 20 min. Thereafter, an appropriate aldehyde (1 eq.) dissolved in MeOH (10 mL) was added, and the resultant reaction mixture allowed to stir for 12 h at room temperature. The reaction progress was monitored by TLC (Hex: EtOAc), which showed the formation of a new spot, while the traces of starting material were still visible. After the completion of the reaction, the product precipitated out of the solution, which was filtered and washed with ice cold water and methanol. In cases where there was no precipitate formed, the product was poured onto water and extracted with ethyl acetate. The organic layer was dried (Na_2_SO_4_), filtered, and evaporated the solvent *in vacuo*. The resulting crude product was purified by a silica gel column chromatography to give products as solids.

*(E)-1-(5-Bromo-2-hydroxyphenyl)-3-(2,5-dimethyl-1-phenyl-1H-pyrrol-3-yl)prop-2-en-1-one***10a**, Yellow crystalline solid. Yield = 24%. m.p. 148 °C–150 °C. ^1^H NMR (300 MHz, CDCl_3_): δ_H_ 13.3 (1H, s, OH), 8.07 (1H, d, *J* = 14.7 Hz, H_αβ_), 8.00 (1H, d, *J* = 2.1 Hz, ArH), 7.53–7.47 (4H, m, ArHs), 7.23–7.20 (2H, m, ArHs), 7.16 (1H, d, *J* = 14.7 Hz, H_αβ_), 6.90 (1H, d, *J* = 9.0 Hz, ArH), 6.41 (1H, s, pyrrole-H), 2.18 (3H, s, CH_3_), 2.04 (3H, s, CH_3_). ^13^C NMR (75 MHz, CDCl_3_): δ_C_ 192.6, 162.50, 140.7, 138.0, 137.7, 136.9, 132.1, 131.7, 129.7, 128.9, 128.1, 121.9, 120.5, 118.0, 112.9, 110.2, 104.4,12.9, 11.2. HRMS (ESI^+^) *m/z* (ESI-HRMS) found 396.0597, calcd for C_21_H_19_NO_2_Br: 396.0594 [M + H]^+^. Anal. Found: C, 62.79; H, 4.13; N, 3.09%. Anal. calcd. C_21_H_18_NO_2_Br·0.5H_2_O: C, 62.23; H, 4.73; N, 3.46%.

*(E)-1-(5-Bromo-2-hydroxyphenyl)-3-(1-(4-fluorophenyl)-2,5-dimethyl-1H-pyrrol-3-yl)prop-2-en-1-one***10b**, Yellow solid. Yield = 39%. m.p. 135 °C-138 °C. ^1^H NMR (400 MHz, CDCl_3_): δ_H_ 13.3 (1H, s, OH), 8.05 (1H, d, *J* = 14.8 Hz, H_αβ_), 7.99 (1H, d, *J* = 2.4 Hz, ArH), 7.51 (1H, dd, *J* = 2.4 and 8.8 Hz, ArH), 7.34–7.26 (4H, m, ArHs), 7.16 (1H, d, *J* = 14.8 Hz, H_αβ_), 6.90 (1H, d, *J* = 9.2 Hz, ArH), 6.40 (1H, s, pyrrole-H), 2.17 (3H, s, CH_3_), 2.03 (3H, s, CH_3_). ^13^C NMR (100 MHz, CDCl_3_): δ_C_ 192.5, 163.8 (2C), 161.2 (2C), 140.5, 138.1, 136.7, 133.6, 132.0, 131.6, 129.8, 121.8, 120.5, 118.1, 116.7, 113.1, 110.3, 104.5, 12.9, 11.2. *m/z* (ESI-HRMS) found 414.0505, calcd for C_21_H_18_BrFNO_2_: 414.0499 [M + H]^+^. Anal. Found: C, 56.92; H, 2.74; N, 2.87%. Anal. calcd. C_21_H_17_NO_2_BrF·2H_2_O: C, 56.01; H, 4.70; N, 3.11%.

*(E)-1-(5-Bromo-2-hydroxyphenyl)-3-(1-(4-chlorophenyl)-2,5-dimethyl-1H-pyrrol-3-yl)prop-2-en-1-one***10c**, Yellow crystalline solid. Yield = 53%. m.p. 127 °C–129 °C. ^1^H NMR (300 MHz, CDCl_3_): δ_H_ 13.3 (1H, s, OH), 8.03 (1H, d, *J* = 14.4 Hz, H_αβ_), 8.00 (1H, d, *J* = 2.4 Hz, ArH), 7.53–7.48 (3H, m, H_αβ_, ArHs), 7.19–7.14 (3H, m, ArHs), 6.90 (1H, d, *J* = 9.0 Hz, ArH), 6.40 (1H, s, pyrrole-H), 2.17 (3H, s, CH_3_), 2.03 (3H, s, CH_3_). ^13^C NMR (75 MHz, CDCl_3_): δ_C_ 192.6, 162.5, 140.4, 138.1, 136.5, 136.2, 134.9, 131.9, 131.7, 130.0, 129.4, 121.9, 120.6, 118.3, 113.4, 110.3, 104.7, 12.9, 11.3. HRMS (ESI^+^) *m/z* (ESI-HRMS) found 430.0200, calcd for C_21_H_18_NO_2_ClBr: 430.0204 [M + H]^+^. Anal. Found: C, 58.15; H, 3.71; N, 3.19%. Anal. calcd. C_21_H_17_NO_2_BrCl: C, 58.56; H, 3.98; N, 3.25%.

*(E)-1-(5-Bromo-2-hydroxyphenyl)-3-(1-(4-bromophenyl)-2,5-dimethyl-1H-pyrrol-3-yl)prop-2-en-1-one***10d**, Yellow crystalline solid. Yield = 45%. m.p. 141 °C–144 °C. ^1^H NMR (300 MHz, CDCl_3_): δ_H_ 13.3 (1H, s, OH), 8.04 (1H, d, *J* = 15.0 Hz, H_αβ_), 7.99 (1H, d, *J* = 2.4 Hz, ArH), 7.65 (2H, d, *J* = 8.7 Hz, ArHs), 7.52 (1H, dd, *J* = 2.4 and 9.0 Hz, ArH), 7.16 (1H, d, *J* = 14.7 Hz, H_αβ_), 7.10 (2H, d, *J* = 8.7 Hz, ArHs), 6.90 (1H, d, *J* = 8.7 Hz, ArH), 6.40 (1H, s, pyrrole-H), 2.18 (3H, s, CH_3_), 2.03 (3H, s, CH_3_). ^13^C NMR (75 MHz, CDCl_3_): δ_C_ 192.4, 162.4, 140.2, 138.0, 136.6, 136.2, 132.8, 131.6, 131.5, 129.6, 122.7, 121.7, 120.4, 118.1, 113.2, 110.1, 104.6, 12.3, 11.0. HRMS (ESI^+^) *m/z* (ESI-HRMS) found 473.9701, calcd for C_21_H_18_NO_2_Br_2_: 473.9699, [M + H]^+^. Anal. Found: C, 52.65; H, 3.19; N, 2.89%. Anal. calcd. C_21_H_17_NO_2_Br_2_·0.5H_2_O: C, 52.08; H, 3.75; N, 2.89%.

*(E)-1-(5-Bromo-2-hydroxyphenyl)-3-(2,5-dimethyl-1-(3-(trifluoromethyl)phenyl)-1H-pyrrol-3-yl)prop-2-en-1-one***10e**, Yellow crystalline solid. Yield = 20%. m.p. 135 °C–137 °C. ^1^H NMR (300 MHz, CDCl_3_): δ_H_ 13.2 (1H, s, OH), 8.04 (1H, d, *J* = 15.0 Hz, H_αβ_), 8.00 (1H, d, *J* = 2.4 Hz, ArH), 7.77–7.75 (1H, m, ArH), 7.67 (1H, d, *J* = 7.8 Hz, Ar), 7.54 – 7.50 (2H, m ArHs), 7.44 (1H, d, *J* = 7.8 Hz, ArH), 7.18 (1H, d, *J* = 14.7 Hz, H_αβ_), 6.91 (1H, d, *J* = 9.0 Hz, ArH), 6.43 (1H, s, pyrrole-H), 2.19 (3H, s, CH_3_), 2.05 (3H, s, CH_3_); ^13^C NMR (75 MHz, CDCl_3_): δ_C_ 192.4, 162.2, 140.2, 138.2, 138.0, 136.0, 131.6, 131.6, 131.4, 130.3, 125.5, 125.2, 124.9, 121.6, 120.4, 118.4, 114.9, 113.6, 110.1, 105.0, 12.8, 11.1. *m/z* (ESI-HRMS) found 464.0456 calcd for C_22_H_16_NO_2_F_3_Br: 464.0468 [M + H]^+^. Anal. Found: C, 57.24; H, 4.76; N, 2.86%. Anal. calcd. C_22_H_17_NO_2_BrF_3_·0.5CH_3_OH: C, 56.27; H, 3.99.; N, 2.92%.

*(E)-1-(5-Bromo-2-hydroxyphenyl)-3-(2,5-dimethyl-1-(4-(trifluoromethyl)phenyl)-1H-pyrrol-3-yl)prop-2-en-1-one***10f**, Yellow crystalline solid. Yield = 70%. m.p. 157 °C–159 °C. ^1^H NMR (400 MHz, CDCl_3_): δ_H_ 13.2 (1H, s, OH), 8.04 (1H, d, *J* = 14.8 Hz, H_αβ_), 8.00 (1H, d, *J* = 2.4 Hz, ArH), 7.80 (2H, d, *J* = 8.4 Hz, ArHs), 7.52 (1H, dd, *J* = 2.4 and 8.8 Hz, ArH), 7.37 (2H, d, *J* = 8.4 Hz, ArHs), 7.18 (1H, d, *J* = 14.8 Hz, H_αβ_), 6.91 (1H, d, *J* = 8.8 Hz, ArH), 6.43 (1H, s, pyrrole-H), 2.19 (3H, s, CH_3_), 2.05 (3H, s, CH_3_). ^13^C NMR (100 MHz, CDCl_3_): δ_C_ 192.6, 162.5, 140.1, 138.2, 136.1, 131.7, 131.3, 130.9, 128.8, 126.9, 125.1, 122.4, 121.81, 120.6, 118.6, 113.8, 110.3, 105.2, 12.9, 11.2. *m/z* (ESI-HRMS) found 464.0468, calcd for C_22_H_18_NO_2_BrF_3_: 464.0468 [M + H]^+^. Anal. Found: C, 56.60; H, 3.88; N, 2.96%. Anal. calcd. C_22_H_17_NO_2_BrF_3_·0.5CH_3_OH: C, 56.27; H, 3.99; N, 2.92%.

*(E)-1-(5-Bromo-2-hydroxyphenyl)-3-(2,5-dimethyl-1-(2-(trifluoromethyl)phenyl)-1H-pyrrol-3-yl)prop-2-en-1-one***10g**, Yellow crystalline solid. Yield = 71%. m.p. 115 °C–118 °C. ^1^H NMR (400 MHz, CDCl_3_): δ_H_ 13.3 (1H, s, H_13_), 8.04 (1H, d, *J* = 14.7 Hz, H_αβ_), 8.00 (1H, d, *J* = 2.4 Hz, ArH), 7.89–7.86 (1H, m, ArH), 7.76–7.71 (1H, m, ArH), 7.68–7.63 (1H, m, ArH), 7.51 (1H, dd, *J* = 2.4 and 9.0 Hz, ArH), 7.32–7.27 (1H, m, ArH), 7.17 (1H, d, *J* = 14.7 Hz, H_αβ_), 6.90 (1H, d, *J* = 8.7 Hz, ArH), 6.41 (1H, s, pyrrole-H), 2.09 (3H, s, CH_3_), 1.95 (3H, s, CH_3_). ^13^C NMR (75 MHz, CDCl_3_): δ_C_ 192.7, 162.5, 140.5, 138.1, 137.8, 135.8, 133.4, 132.9, 131.7, 131.3, 129.9, 129.0, 127.8, 121.8, 120.5, 118.3, 113.4, 110.4, 110.3, 104.4, 12.5, 10.9. *m/z* (ESI-HRMS) found 462.0299, calcd for C_22_H_16_NO_2_F_3_Br: 462.0322 [M-H]^+^. Anal. Found: C, 57.20; H, 1.94; N, 2.95%. Anal. calcd. C_22_H_17_NO_2_BrF_3_: C, 56.91; H, 3.69; N, 3.02%.

*(E)-1-(5-Bromo-2-hydroxyphenyl)-3-(2,5-dimethyl-1-(4-nitrophenyl)-1H-pyrrol-3-yl)prop-2-en-1-one***10h**, Yellow crystalline solid. Yield = 37%. m.p. 168 °C–170 °C. ^1^H NMR (300 MHz, CDCl_3_): δ_H_ 13.2 (1H, s, OH), 8. 41 (2H, d, *J* = 8.8 Hz, ArHs), 8.04–7.99 (2H, m, ArH_,_ H_αβ_), 7.53 (1H, dd, *J* = 2.4 and 8.8 Hz, ArH), 7.43 (2H, d, *J* = 8.8 Hz, ArHs), 7.19 (1H, d, *J* = 14.8 Hz, H_αβ_), 6.91 (1H, d, *J* = 8.8 Hz, ArH), 6.45 (1H, s, pyrrole-H), 2.21 (3H, s, CH_3_), 2.10 (3H, s, CH_3_). ^13^C NMR (75 MHz, CDCl_3_): δ_C_ 192.6, 162.5, 147.6, 143.3, 139.6, 138.3, 135.6, 131.7, 131.4, 129.1, 125.2, 121.7, 120.6, 119.0, 114.2, 110.3, 105.7, 13.0, 11.3. *m/z* found 441.0444, calcd for C_21_H_18_N_2_O_4_Br: 441.0444 [M + H]^+^.

*(E)-1-(5-bromo-2-hydroxyphenyl)-3-(2,5-dimethyl-1-(3-nitrophenyl)-1H-pyrrol-3-yl)prop-2-en-1-one***10i**, Yellow crystalline solid. Yield = 68%. m.p. 102 °C–104 °C. ^1^H NMR (300 MHz, CDCl_3_): δ_H_ 13.2 (1H, s, OH), 8.39 – 8.35 (1H, m, ArH), 8.14 (1H, t, *J* = 2.1 Hz, ArH), 8.06–8.00 (2H, m, ArH, H_αβ_), 7.75 (1H, t, *J* = 7.8 Hz, Ar), 7.62–7.58 (1H, m, ArH), 7.53 (1H, dd, *J* = 2.4 and 9.0 Hz, ArHs), 7.20 (1H, d, *J* = 15.0 Hz, H_αβ_), 6.91 (1H, d, *J* = 9.0 Hz, ArH), 6.45 (1H, s, pyrrole-H), 2.21 (3H, s, CH_3_), 2.07 (3H, s, CH_3_); ^13^C NMR (75 MHz, CDCl_3_): δ_C_ 192.6, 162.5, 148.9, 139.9, 138.9, 138.3, 135.9, 134.3, 131.7, 131.6, 130.7, 123.8, 123.4, 121.7, 120.6, 118.7, 114.1, 110.3, 105.4, 12.9, 11.3. *m/z* (ESI-HRMS) found 441.0278, calcd for C_21_H_18_N_2_O_4_Br: 441.0444 [M + H]^+^. Anal. Found: C, 57.66; H, 4.16; N, 6.10%. Anal. calcd. C_21_H_17_N_2_O_4_Br: C, 57.16; H, 3.88; N, 6.35%.

*(E)-1-(5-Bromo-2-hydroxyphenyl)-3-(1-(4-methoxyphenyl)-2,5-dimethyl-1H-pyrrol-3-yl)prop-2-en-1-one***10j**, Yellow crystalline solid. Yield = 14%. m.p. 138 °C–140 °C. ^1^H NMR (400 MHz, CDCl_3_): δ_H_ 13.3 (1H, s, OH), 8.07 (1H, d, *J* = 14.8 Hz, H_αβ_), 8.00 (1H, d, *J* = 2.4 Hz, ArH), 7.51 (1H, dd, *J* = 2.4 and 8.8 Hz, ArH), 7.15 (1H, d, *J* = 14.8 Hz, H_αβ_), 7.12 (2H, d, *J* = 8.8 Hz, ArHs), 7.01 (2H, d, *J* = 8.8 Hz, ArHs), 6.90 (1H, d, *J* = 8.8 Hz, ArH), 6.38 (1H, s, pyrrole-H), 3.87 (3H, s, OCH_3_), 2.17 (3H, s, CH_3_), 2.02 (3H, s, CH_3_). ^13^C NMR (100 MHz, CDCl_3_): δ_C_ 192.4, 162.5, 159.8, 140.9, 138.1, 137.3, 132.3, 131.7, 130.2, 129.1, 121.8, 120.6, 117.8, 114.9, 112.7, 110.0, 104.0, 55.6, 12.8, 11.1. *m/z* (ESI-HRMS) found 426.0698, calcd for C_22_H_21_NO_3_Br: 426.0699 [M + H]^+^.

*(E)-1-(5-Bromo-2-hydroxyphenyl)-3-(2,5-dimethyl-1H-pyrrol-3-yl)prop-2-en-1-one***10k**, Orange solid. Yield = 9%. m.p. 88 °C–89 °C. ^1^H NMR (400 MHz, CDCl_3_): δ_H_ 13.3 (1H, s, OH), 8.00–7.97 (3H, m, ArHs, H_αβ_), 7.50 (1H, dd, *J* = 2.4 and 8.8 Hz, ArH), 7.08 (1H, d, *J* = 14.8 Hz, H_αβ_), 6.89 (1H, d, *J* = 8.8 Hz, ArH), 6.22 (1H, s, pyrrole-H), 2.39 (3H, s, CH_3_), 2.25 (3H, s, CH_3_). ^13^C NMR (100 MHz, CDCl_3_): δ_C_ 192.7, 162.3, 140.6, 137.9, 134.6, 131.6, 128.9, 121.8, 120.6, 118.6, 112.9, 110.2, 104.2, 12.9, 11.5. *m/z* (ESI-HRMS) found 318.0144, calcd for C_15_H_13_NO_2_Br: 318.0130 [M-H]^+^. Anal. Found: C, 55.69; H, 2.48; N, 3.44%; Anal. calcd. C_15_H_14_NO_2_Br·CH_3_OH: C, 55.37; H, 4.80; N, 4.17%.

*(E)-1-(5-Bromo-2-hydroxyphenyl)-3-(1-cyclohexyl-2,5-dimethyl-1H-pyrrol-3-yl)prop-2-en-1-one***10l**, Yellow solid. Yield = 42%. m.p. 73 °C–76 °C. ^1^H NMR (400 MHz, CDCl3): δ_H_ 13.4 (1H, s, OH), 8.03 (1H, d, *J* = 14.4 Hz, H_αβ_), 7.96 (1H, d, *J* = 2.4 Hz, ArH), 7.49 (1H, dd, *J* = 2.4 and 9.2 Hz, ArH), 7.07 (1H, d, *J* = 14.4 Hz, H_αβ_), 6.88 (1H, d, *J* = 8.8 Hz, ArH), 6.27 (1H, s, pyrrole-H), 4.00–3.92 (1H, m, cyclic-H), 2.44 (3H, s, CH_3_), 2.33 (3H, s, CH_3_), 2.00–1.75 (7H, m, cyclic-Hs), 1.45–1.20 (3H, m, cyclic-Hs). ^13^C NMR (100 MHz, CDCl_3_): δ_C_ 192.6, 162.5, 140.7, 137.9, 136.2, 131.5, 131.1, 122.0, 120.4, 112.0, 110.2, 105.5, 57.4, 32.3 (2C), 26.4, 25.9, 14.3, 11.8. *m/z* (ESI-HRMS) found 402.1061, calcd for C_21_H_25_NO_2_Br: 402.1063 [M + H]^+^. Anal. Found: C, 62.08; H, 5.06; N, 3.44%; Anal. calcd. C_21_H_24_NO_2_Br: C, 62.69; H, 6.01; N, 3.48%.

*(E)-1-(5-Bromo-2-hydroxyphenyl)-3-(1H-imidazol-4-yl)prop-2-en-1-one***11a**. Yellow solid. Yield = 9%. m.p. 116 °C–120 °C. ^1^H NMR (600 MHz, DMSO-*d_6_*): δ_H_ 12.3 (1H, s, OH), 8.01 (1H, d, *J* = 2.4 Hz, ArH), 7.88 (1H, s, imidazole-H), 7.73 (1H, s, imidazole-H), 7.70 (1H, d, *J* = 15.0 Hz, H_αβ_), 7.64–7.61 (2H, m, ArH, H_αβ_), 6.94 (1H, d, *J* = 8.4 Hz, ArH); ^13^C NMR (150 MHz, DMSO-*d_6_*): δ_C_ 191.9, 159.9, 138.5, 137.7, 136.7, 131.9 (2C), 123.4, 120.1 (2C), 117.8, 110.13. *m/z* (ESI-HRMS) found 292.9914, calcd for C_12_H_10_N_2_O_2_Br: 292.9920 [M + H]^+^. Anal. Found: C, 49.99; H, 3.30; N, 9.76. Anal. calcd. C_12_H_9_N_2_O_2_Br: C, 49.17; H, 3.09; N, 9.56%.

*(E)-3-(2,5-Dimethyl-1-(4-nitrophenyl)-1H-pyrrol-3-yl)-1-phenylprop-2-en-1-one,***12**, Yellow crystalline solid. Yield = 65%. m.p. 169 °C–174 °C. ^1^H NMR (300 MHz, CDCl_3_): δ_H_ 8.39 (2H, d, *J* = 9.0 Hz, ArHs), 8.03 – 8.00 (2H, m, ArHs), 7.89 (1H, d, *J* = 15.3 Hz, H_αβ_), 7.57–7.46 (3H, m, ArHs), 7.40 (2H, d, *J* = 8.7 Hz, ArHs), 7.21 (1H, d, *J* = 15.0 Hz, H_αβ_), 6.39 (1H, s, pyrrole-H), 2.18 (3H, s, CH_3_), 2.07 (3H, s, CH_3_). ^13^C NMR (75 MHz, CDCl_3_): δ_C_ 190.7, 147.6, 143.6, 139.2, 137.9, 134.1, 132.4, 130.9, 129.2, 128.6, 128.4, 125.1, 118.9, 117.4, 105.7, 13.1, 11.3. *m/z* (ESI-HRMS) found 347.1392, calcd for C_21_H_19_N_2_O_3_: 347.1390 [M + H]^+^. Anal. Found: C, 72.30; H, 6.22; N, 7.44%. Anal. calcd. C_21_H_18_N_2_O_3_: C, 72.82; H, 5.24; N, 8.09%.

*(E)-3-(2,5-Dimethyl-1-(4-nitrophenyl)-1H-pyrrol-3-yl)-1-(p-tolyl)prop-2-en-1-one,***13**, Yellow solid. Yield = 34%. m.p. 172 °C–173 °C. ^1^H NMR (400 MHz, CDCl_3_): δ_H_ 8.39 (2H, d, *J* = 8.8 Hz, ArHs), 7.92 (2H, d, *J* = 8.4 Hz, ArHs), 7.88 (1H, d, *J* = 15.2 Hz, H_αβ_), 7.41 (2H, d, *J* = 8.8 Hz, ArHs), 7.28 (2H, d, *J* = 8 Hz, ArHs), 7.21 (1H, d, *J* = 15.2 Hz, H_αβ_), 6.40 (1H, s, pyrrole-H), 2.43 (3H, s, CH_3_), 2.18 (3H, s, CH_3_), 2.06 (3H, s, CH_3_). ^13^C NMR (75 MHz, CDCl_3_): δ_C_ 190.2, 147.5, 143.7, 143.1, 137.4, 136.6, 133.9, 130.8, 129.3, 129.2, 128.6, 125.0, 118.9, 117.4, 105.7, 21.9, 13.1, 11.3. *m/z* (ESI-HRMS) found 361.1552, calcd for C_22_H_21_N_2_O_3_: 361.1547 [M + H]^+^. Anal. Found: C, 73.08; H, 5.86; N, 7.43%. Anal. calcd. C_22_H_20_N_2_O_3_: C, 73.32; H, 5.59; N, 7.77%.

*(E)-3-(2,5-Dimethyl-1-(4-nitrophenyl)-1H-pyrrol-3-yl)-1-(4-fluorophenyl)prop-2-en-1-one,***14**, Yellow solid. Yield = 86%. m.p. 160 °C–164 °C. ^1^H NMR (300 MHz, CDCl_3_): δ_H_ 8.39 (2H, d, *J* = 9.0 Hz, ArHs), 8.07–8.02 (2H, m, ArHs), 7.89 (1H, d, *J* = 15.3 Hz, H_αβ_), 7.41 (2H, d, *J* = 9.0 Hz, ArHs), 7.205–7.12 (3H, m, ArHs_,_ H_αβ_), 6.39 (1H, s, pyrrole-H), 2.19 (3H, s, CH_3_), 2.07 (3H, s, CH_3_). ^13^C NMR (75 MHz, CDCl_3_): δ_C_ 189.1, 147.7, 143.8, 138.3, 135.6, 134.5, 131.1, 131.0, 129.3, 125.2, 119.1, 117.0, 115.9, 115.7, 105.7, 13.2, 11.4. *m/z* (ESI-HRMS) found 365.1305, calcd for C_21_H_18_N_2_O_3_F: 365.1296 [M + H]^+^. Anal. Found: C, 69.15; H, 5.24; N, 7.19%. Anal. calcd. C_21_H_17_N_2_O_3_F: C, 69.22; H, 4.70; N, 7.69%.

*(E)-3-(2,5-Dimethyl-1-(4-nitrophenyl)-1H-pyrrol-3-yl)-1-(4-methoxyphenyl)prop-2-en-1-one,***15**, Yellow crystalline solid. Yield = 18%. m.p. 157 °C–158 °C. ^1^H NMR (300 MHz, CDCl_3_): δ_H_ 8.39 (2H, d, *J* = 8.7 Hz, ArHs), 8.04 (2H, d, *J* = 9.0 Hz, ArHs), 7.88 (1H, d, *J* = 15.3 Hz, H_αβ_), 7.41 (2H, d, *J* = 9.0 Hz, ArHs), 7.23 (1H, d, *J* = 15.3 Hz, H_αβ_), 6.97 (2H, d, *J* = 9.0 Hz, ArHs), 6.39 (1H, s, pyrrole-H), 3.89 (3H, s, CH_3_), 2.18 (3H, s, CH_3_), 2.07 (3H, s, CH_3_). ^13^C NMR (75 MHz, CDCl_3_): δ_C_ 189.0, 163.1, 147.4, 143.7, 137.0, 133.8, 131.9, 130.7, 130.6, 129.1, 125.0, 118.9, 117.1, 113.8, 105.5, 55.5, 13.0, 11.5. *m/z* (ESI-HRMS) found 377.1502, calcd for C_22_H_21_N_2_O_4_: 377.1496 [M + H]^+^. Anal. Found: C, 68.91; H, 5.73; N, 7.23%. Anal. calcd. C_22_H_20_N_2_O_4_·0.5CH_3_OH: C, 68.86; H, 5.65; 7.14%.

*(E)-1-(2,4-Dimethoxyphenyl)-3-(2,5-dimethyl-1-(4-nitrophenyl)-1H-pyrrol-3-yl)prop-2-en-1-one***16**. Yellow crystalline solid. Yield = 21%. m.p. 146 °C–149 °C. ^1^H NMR (600 MHz, CDCl_3_): δ_H_ 8.38 (2H, d, *J* = 8.4 Hz, ArHs), 7.75–7.72 (2H, m, ArH_,_ H_αβ_), 7.40 (2H, d, *J* = 9.0 Hz, ArHs), 7.16 (1H, d, *J* = 15.6 Hz, H_αβ_), 6.55 (1H, dd, *J* = 2.4 and 9.0 Hz, ArH), 6.50 (1H, d, *J* = 2.4 Hz, ArH), 6.32 (1H, s, pyrrole-H), 3.90 (3H, s, CH_3_), 3.87 (3H, s, CH_3_), 2.15 (3H, s, CH_3_), 2.053 (3H, s, CH_3_). ^13^C NMR (75 MHz, CDCl_3_): δ_C_ 191.0, 163.8, 160.2, 147.5, 143.8, 135.6, 133.2, 132.7, 130.5, 129.2, 125.0, 123.2, 122.9, 119.1, 105.8, 105.1, 98.9, 55.9, 55.7, 13.1, 11.3. *m/z* (ESI-HRMS) found 407.1608, calcd for C_23_H_23_N_2_O_5_: 407.1601 [M + H]^+^. Anal. Found C, 67.99; H, 5.84; N, 6.86%; Anal. calcd. C_23_H_22_N_2_O_5_: C, 67.41; H, 5.61; N, 6.61%.

*(E)-3-(2,5-Dimethyl-1-(4-nitrophenyl)-1H-pyrrol-3-yl)-1-(4-nitrophenyl)prop-2-en-1-one,***17**, Reddish solid. Yield = 5%. m.p. 175 °C–178 °C. ^1^H NMR (400 MHz, CDCl_3_): δ_H_ 8.41 (2H, d, *J* = 8.8 Hz, ArHs), 8.33 (2H, d, *J* = 8.4 Hz, ArHs), 8.13 (2H, d, *J* = 8.8 Hz, ArHs), 7.93 (1H, d, *J* = 15.2 Hz, H_αβ_), 7.42 (2H, d, *J* = 8.8 Hz, ArHs), 7.14 (1H, d, *J* = 15.2 Hz, H_αβ_), 6.40 (1H, s, pyrrole-H), 2.20 (3H, s, CH_3_), 2.07 (3H, s, CH_3_). ^13^C NMR (100 MHz, CDCl_3_): δ_C_ 188.9, 149.9, 147.7, 144.3, 143.4, 139.9, 135.3, 131.4 (2C), 129.3, 129.2, 125.2, 123.9, 118.9, 116.5, 105.6, 13.1, 11.4. *m/z* (ESI-HRMS) found 392.1246, calcd for C_21_H_18_N_3_O_5_: 392.1241 [M + H]^+^. Anal. Found: C, 63.30; H, 5.00; N, 9.29%. Anal. calcd. C_21_H_17_N_3_O_5_·0.5CH_3_COCH_2_CH_3_: C, 63.44; H, 4.86; N, 9.65%.

*(E)-1-(5-Chloro-2-hydroxyphenyl)-3-(2,5-dimethyl-1-(3-(trifluoromethyl)phenyl)-1H-pyrrol-3-yl)prop-2-en-1-one***18**. Yellow crystalline solid. Yield = 87%. m.p. 110 °C–112 °C. ^1^H NMR (400 MHz, CDCl_3_): δ_H_ 13.2 (1H, s, OH), 8.04 (1H, d, *J* = 2.4 Hz, ArH), 7.76 (1H, d, *J* = 7.6 Hz, ArH), 7.70–7.66 (1H, m, ArH), 7.52–7.49 (1H, m, ArH), 7.45–7.42 (1H, m, ArH), 7.40 (1H, dd, *J* = 2.4 and 9.2 Hz, ArH), 7.19 (1H, d, *J* = 14.8 Hz, H_αβ_), 6.95 (1H, d, *J* = 8.8 Hz, ArH), 6.42 (1H, s, pyrrole-H), 2.19 (3H, s, CH_3_), 2.05 (3H, s, CH_3_). ^13^C NMR (100 MHz, CDCl_3_): δ_C_ 192.7, 162.1, 140.1, 138.4, 136.2, 135.9, 135.4, 132.3, 131.7, 131.6, 130.4, 128.7, 125.7, 125.1, 123.3, 121.2, 120.2, 118.5, 113.8, 105.1, 12.9, 11.2. *m/z* (ESI-HRMS) found 420.0945, calcd for C_22_H_17_NO_2_ClF_3_: 420.0948 [M + H]^+^. Anal. Found: C, 62.34; H, 4.28; N, 2.94%; Anal. calcd. C_22_H_17_NO_2_ClF_3_·0.5CH_3_CH_2_OH: C, 62.38; H, 4.55; N, 3.16%.

*(E)-1-(5-Chloro-2-hydroxyphenyl)-3-(2,5-dimethyl-1-(3-nitrophenyl)-1H-pyrrol-3-yl)prop-2-en-1-one***19**, Yellow crystalline solid. Yield = 89%. m.p. 71 °C–74 °C. IR (cm^−1^): 1630 (C=O), 1527 (C=C). ^1^H NMR (300 MHz, CDCl_3_): δ_H_ 13.2 (1H, s, OH), 8.38 – 8.34 (1H, m, ArH), 8.14 – 8.13 (1H, m, ArH), 8.03 (1H, d, *J* = 15.0 Hz, H_αβ_), 7.86 (1H, d, *J* = 2.7 Hz, ArH), 7.77–7.72 (1H, m, ArH), 7.61–7.58 (1H, m, ArH), 7.40 (1H, dd, *J* = 2.4 and 8.7 Hz, ArH), 7.20 (1H, d, *J* = 14.7 Hz, H_αβ_), 6.96 (1H, d, *J* = 9.0 Hz, ArH), 6.44 (1H, s, pyrrole-H), 2.19 (3H, s, CH_3_), 2.07 (3H, s, CH_3_). ^13^C NMR (75 MHz, CDCl_3_): δ_C_ 192.8, 162.1, 148.9, 139.8, 138.9, 135.9, 135.5, 134.3, 131.5, 130.7, 128.7, 123.7, 123.3 (2C), 121.1, 120.2, 118.7, 114.1, 105.3, 13.0, 11.3. *m/z* (ESI-HRMS) found 397.0947, calcd for C_21_H_18_N_2_O_4_Cl: 397.0955 [M + H]^+^. Anal. Found: C, 63.74; H, 5.23; N, 6.62%; Anal. calcd. C_21_H_17_N_2_O_4_Cl·0.4 CH_3_CH_2_OH: C, 63.06; H, 4.71; N, 6.75%.

### 3.3. X-ray Crystallographic Analysis

A single crystal was covered in a small amount of paratone N oil and mounted on a MiTeGen microloop. X-ray intensity data were collected at 100 K on a Bruker DUO APEX CCD with graphite monochromated Mo radiation (λ = 0.71073). The detector to crystal distance was 60 mm. Data were collected using phi and omega scans and were scaled and reduced using the APEX III software suite (Bruker SAINT). The structure was solved using SHELXT 2018/2 (Sheldrick, 2018) and refined using SHELXL-2018/3 (Sheldrick, 2018) [52]. Hydrogen atoms were placed in calculated positions and refined using the riding model. The program X-SEED [53], an interface to SHELX, was used during the structure solution and refinement. The accession code for **10e** is (CCDC 1991652). The supplementary information contains crystallographic data for compound **10e**. The data can be obtained free of charge from The Cambridge Crystallographic Data Centre on http://www.ccdc.cam.ac.uk/conts/retrieving.html or The Director, CCDC, 12, Union Road, Cambridge CB2 1EZ, UK; fax: +44 1223 336033; or deposit@ccdc.cam.ac.uk.

### 3.4. In Vitro Antitrypanosomal Assay

*Trypanosoma brucei brucei* 427 trypomastigotes were cultured in Iscove’s Modified Dulbecco’s medium (IMDM, Lonza, Basel Switzerland) and supplemented with 10% fetal calf serum, HMI-9 supplement [54], hypoxanthine and penicillin/streptomycin at 37 °C in a 5% CO_2_ incubator. Serial dilutions of test compounds were incubated with the parasites in 96-well plates for 24 h, and residual parasite viability in the wells determined by adding 20 µL resazurin toxicology reagent (Sigma-Aldrich) and incubating for an additional 24 h. Reduction of resazurin to resorufin by viable parasites was assessed by fluorescence readings (excitation 560 nm, emission 590 nm) in a Spectramax M3 plate reader (Molecular Devices, San Jose, CA, USA). Fluorescence readings were converted to % parasite viability relative to the average readings obtained from untreated control wells. IC_50_ values were determined by plotting % viability vs. log[compound] and performing non-linear regression using GraphPad Prism (v. 5.02) software [55].

### 3.5. In Vitro Cytotoxicity Assay

HeLa cells (Cellonex, Johannesburg, South Africa) seeded in 96-well plates were incubated with 20 µM test compounds and cell viability assessed with resazurin, as previously described [56]. Fluorescence readings (excitation 560 nm, emission 590 nm) obtained for the individual wells were converted to % cell viability relative to the average readings obtained from untreated control wells. Plots of % cell viability vs. log[compound] were used to determine IC_50_ values by non-linear regression using GraphPad Prism (v. 5.02) [56].

## 4. Conclusions

In this study, we presented a series of arylpyrrole-chalcone derivatives, which were obtained in yields ranging from poor to excellent. Analysis of the NMR and crystal structure of compound **10f** confirmed unequivocally that achieved compounds assumed a *trans* configuration about the double bond of the α,β-unsaturated carbonyl system. The in vitro bioassays data of resultant compounds suggested that they are generally non-toxic, and displayed weak growth inhibition of HeLa cells at 20 µM, and none of the compounds inhibited cell viability to <80%. More importantly, these compounds showed anti-trypanosomal activity, with most active compounds **10e** and **10h** displaying IC_50_ values of < 6.0 µM. Despite modest activity, a combination of electron donating groups on the ring A and electron withdrawing groups on the B ring appeared to be required for the activity of these compounds. Compounds lacking the aryl groups on the B ring side of the chalcone scaffold showed no activity at the maximum tested concentration, suggesting that the arylpyrrole moiety is necessary for activity, and it is worthy of further exploitation for the development of alternative compounds for the treatment of infections caused by trypanosomes.

## Figures and Tables

**Table 1:** In vitro bioassay data for chalcone derivatives **10a**–**l**, **11a**–**b**, and **12**–**19** showing IC_50_ values for inhibition of *T. b. brucei* 427.

Comp	*T. b. brucei*	Comp	*T. b. brucei*
	IC_50_/µM		IC_50_/µM
**10a**	11.5	10l	11.5
**10b**	6.9	11a	na
**10c**	40.0	11b	9.1
**10d**	10.5	12	na
**10e**	4.1	13	44.3
**10f**	11.4	14	36.5
**10g**	10.0	15	12.3
**10h**	5.1	16	10.6
**10i**	9.3	17	na
**10j**	na	18	9.3
**10k**	na	19	11.7
**PMD**	0.68 nM	-	-

PMD = Pentamidine. na = not active (compounds producing a cell viability of ≥50% in the preliminary single point assay at 20 μM).

## Supplementary Materials

The following are available online (Synthesis of arylpyrrole intermediates, compound **8a-l**, X-ray crystallographic data of compound **10e** and selected ^1^H and ^13^C NMR spectra of some compounds).
