# Supplementary material for: Synthesis, Structure and In Vitro Anti-Trypanosomal Activity of Non-Toxic Arylpyrrole-Based Chalcone Derivatives
Source: Molecules. 2020 Apr 4;25(7):1668. doi: 10.3390/molecules25071668 (PMC7181280; doi:10.3390/molecules25071668)
Supplement: Supplementary file 1 [file molecules-25-01668-s001.pdf]

Synthesis, Structure and In Vitro Anti-Trypanosomal Activity of Non-Toxic Arylpyrrole-Based Chalcone Derivatives

Ayanda I. Zulu [1], Ogunyemi O. Oderinlo [1], Cuan Kruger [1], Michelle Isaacs [2], Heinrich C. Hoppe [2,3], Vincent J. Smith [1,2], Clinton G.L. Veale [4,5] and Setshaba D. Khanye [1,2,5,*]

[1] Department of Chemistry, Faculty of Science, Rhodes University, Grahamstown 6140, South Africa; azulu50@gmail.com (A.I.Z.); oderinloyemi@yahoo.com (O.O.O.); g13k8200@campus.ru.ac.za (C.K.); v.smith@ru.ac.za (V.J.S.)
[2] Centre for Chemico and Biomedicinal Research, Rhodes University, Grahamstown 6140, South Africa; m.isaacs@ru.ac.za (M.I.); h.hoppe@ru.ac.za (H.C.H.)
[3] Department of Biochemistry and Microbiology, Faculty of Science, Rhodes University, Grahamstown 6140, South Africa
[4] School of Chemistry and Physics, Pietermaritzburg Campus, University of KwaZulu-Natal, Private Bag X01, Scottsville 3209, South Africa; VealeC@ukzn.ac.za
[5] Division of Pharmaceutical Chemistry, Faculty of Pharmacy, Rhodes University, Grahamstown 6140, South Africa
* Correspondence: s.khanye@ru.ac.za; Tel.: +27-46-603-8397

Academic editor: Andrew Tsotinis
Received: 21 March 2020; Accepted: 02 April 2020; Published: date

1. SYNTHESIS

1.1. General procedure for the synthesis of arylpyrroles

Aniline (1.1 eq.) and 2,5-hexanedione (1 eq.) were mixed and heated to 160 °C. The reaction was allowed to stir for 16 h. The TLC showed a mixture of spots including desired products and some starting material. After cooling, the reaction product solidified and it was dissolved in ethanol (15 mL) before adding, silica gel to prepare a dry load column. The crude product was then purified by silica gel column chromatography to desired arylpyrrole as solids.

2,5-Dimethyl-1-phenyl-1*H*-pyrrole

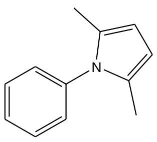

Dark brown crystalline solid (68%). ^{1}HNMR (600 MHz, $CDCl_3$): δ_H 7.48 (2H, t, *J* = 7.8 Hz, ArHs), 7.42 (1H, t, *J* = 7.2 Hz, ArH), 7.48 (2H, d, *J* = 7.2 Hz, ArHs), 5.91 (2H, s, pyrrole-Hs), 2.06 (6H, s, CH_3).

1-(4-Fluorophenyl)-2,5-dimethyl-1*H*-pyrrole

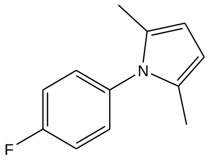

Tan crystalline solid (38%). ^{1}H NMR (600 MHz, $CDCl_3$): δ_H 7.20 – 7.18 (2H, m, ArHs), 7.17 – 7.14 (2H, m, ArHs), 5.91 (2H, s, pyrrole-Hs), 2.03 (6H, s, CH_3).

1-(4-Chlorophenyl)-2,5-dimethyl-1*H*-pyrrole

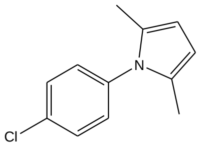

Tan crystalline solid (81%). ^{1}H NMR (300 MHz, $CDCl_3$): δ_H 7.46 – 7.42 (2H, m, ArHs), 7.18 – 7.13 (2H, m, ArHs), 5.91 (2H, s, pyrrole-Hs), 2.03 (6H, s, CH_3).

1-(4-Bromophenyl)-2,5-dimethyl-1*H*-pyrrole

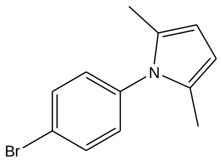

Brown crystalline solid (60%). ^{1}H NMR (300 MHz, $CDCl_3$): δ_H 7.59 (2H, d, J = 8.7 Hz, ArHs), 7.09 (2H, d, J = 8.7 Hz, ArHs), 5.90 (2H, s, pyrrole-Hs), 2.02 (6H, s, CH_3).

2,5-Dimethyl-1-(4-nitrophenyl)-1*H*-pyrrole

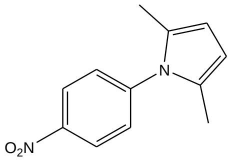

Yellow crystalline solid (70%). ^{1}H NMR (300 MHz, $CDCl_3$): δ_H 8.35 (2H, d, J = 9.0 Hz, ArHs), 7.39 (2H, d, J = 9.0 Hz, ArHs), 5.95 (2H, s, pyrrole-Hs), 2.07 (6H, s, CH_3).

2,5-Dimethyl-1-(3-nitrophenyl)-1*H*-pyrrole

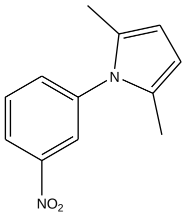

Yellow crystalline solid (65%). ^{1}H NMR (400 MHz $CDCl_3$): δ_H 8.29 – 8.26 (1H, m, ArH), 8.12 – 8.11 (1H, m, ArHs), 7.69 – 7.65 (1H, m, ArHs), 7.59 – 7.56 (1H, m, ArHs), 5.95 (2H, s, pyrrole-Hs), 2.06 (6H, s, CH_3).

2,5-Dimethyl-1-(4-(trifluoromethyl)phenyl)-1*H*-pyrrole

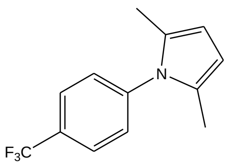

White crystalline solid (72 %). ^{1}H NMR (600 MHz, $CDCl_3$): δ_H 7.75 (2H, d, J = 8.4 Hz, ArHs), 7.35 (2H, d, J = 7.8 Hz, ArHs), 5.94 (2H, s, pyrrole-Hs), 2.06 (6H, s, CH_3).

2,5-Dimethyl-1-(3-(trifluoromethyl)phenyl)-1*H*-pyrrole

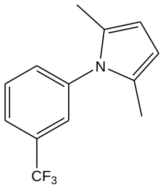

White crystalline solid (67%). ^{1}H NMR (300 MHz, $CDCl_3$): δ_H 7.70 - 7.65 (1H, m, H_1), 7.61 (1H, t, *J* = 7.5, 7.8 Hz, ArH), 7.51 (1H, s, ArH), 7.43 (1H, d, *J* = 7.5 Hz, ArH), 5.94 (2H, s, pyrrole-Hs), 2.05 (6H, s, CH_3).

2,5-Dimethyl-1-(2-(trifluoromethyl)phenyl)-1*H*-pyrrole

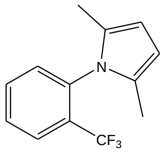

White crystalline solid (55%). ^{1}H NMR (300 MHz, $CDCl_3$): δ_H 7.84 – 7.81 (1H, m, ArH), 7.70 – 7.64 (1H, m, ArH), 7.60 – 7.55 (1H, m, ArHs) 7.28 – 7.25 (1H, m, ArH), 5.91 (2H, s, pyrrole-Hs), 1.92 (6H, s, CH_3).

1-(4-Methoxyphenyl)-2,5-dimethyl-1*H*-pyrrole

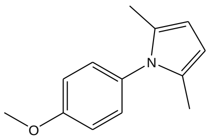

Greyish crystals (58 %). ^{1}H NMR (400 MHz, $CDCl_3$): δ_H 7.13 (2H, d, *J* = 8 Hz, ArHs), 6.97 (2H, d, *J* = 8 Hz, ArHs), 5.88 (2H, s, pyrrole-Hs), 3.86 (3H, s, OCH_3), 2.02 (6H, s, CH_3).

Cyclohexyl-2, 5-dimethyl-1H-pyrrole

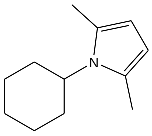

Whitish crystalline solid (70%). ^{1}H NMR (300 MHz, $CDCl_3$): δ_H 5.74 (2H, s, pyrrole-Hs), 3.95 – 3.85 (1H, m, cyclic-H), 2.30 (6H, s, CH_3), 1.99 – 1.71 (7H, m, cyclic-Hs), 1.45 – 1.17 (3H, m, cyclic-Hs).

General procedure for the synthesis of arylpyrrolecarbaldehyde derivatives, 8a - l

To an ice-cold solution of dry DMF (5 mL) under nitrogen atmosphere, $POCl_3$ (1.2 eq) was added dropwise. The reaction mixture was allowed to stir for 20 min, and thereafter an appropriate starting arylpyrrole (1 eq) dissolved in DMF (2.5 mL) was added. The reaction mixture was allowed to warm to room temperature and heated to 60 °C for 4 h. The reaction progress was monitored by TLC, which showed a new spot and the disappearance of the starting material. Thereafter, the reaction product was cooled to the room temperature, poured into crushed ice and quenched with a 20% NaOH resulting in formation of the precipitate. The precipitate was filtered, washed with cold water and dried under fume hood.

2,5-Dimethyl-1-phenyl-1*H*-pyrrole-3-carbaldehyde, 8a

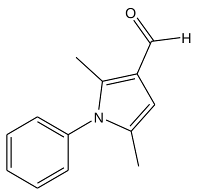

Light brown solid (84%). ^{1}H NMR (300 MHz, $CDCl_3$): δ_H 9.86 (1H, s, CHO), 7.55 – 7.47 (3H, m, ArHs), 7.22 – 7.17 (2H, m, ArHs), 6.38 (1H, s, pyrrole-H), 2.27 (3H, s, CH_3), 1.98 (3H, s, CH_3).

1-(4-Fluorophenyl)-2,5-dimethyl-1*H*-pyrrole-3-carbaldehyde, 8b

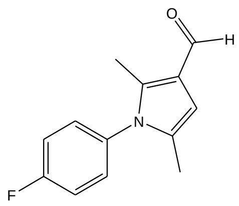

Brown solid (95%). ^{1}H NMR (300 MHz, $CDCl_3$): δ_H 9.85 (1H, s, CHO), 7.24 – 7.16 (4H, m, ArHs), 6.37 (1H, s, pyrrole-H), 2.26 (3H, s, CH_3), 1.97 (3H, s, CH_3).

1-(4-Chlorophenyl)-2,5-dimethyl-1*H*-pyrrole-3-carbaldehyde, 8c

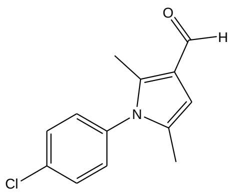

Dark brown solid (93%). ^{1}H NMR (600 MHz, $CDCl_3$): δ_H 9.87 (1H, s, CHO), 7.50 – 7.15 (4H, m, ArHs), 6.38 (1H, s, pyrrole-H), 2.27 (3H, s, CH_3), 1.98 (3H, s, CH_3).

1-(4-Bromophenyl)-2,5-dimethyl-1*H*-pyrrole-3-carbaldehyde, 8d

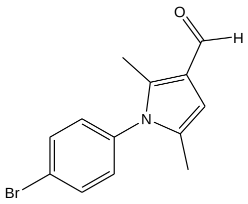

Brown solid (64%). ^{1}H NMR (300 MHz, $CDCl_3$): δ_H 9.86 (1H, s, CHO), 7.66 (2H, d, J = 8.4 Hz, ArHs), 7.09 (2H, d, J = 8.4 Hz, ArHs), 6.38 (1H, s, pyrrole-H), 2.27 (3H, s, CH_3), 1.98 (3H, s, CH_3).

2,5-Dimethyl-1-(4-nitrophenyl)-1*H*-pyrrole-3-carbaldehyde, 8e

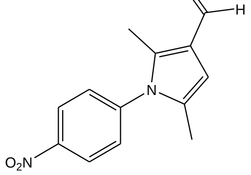

Yellow solid (73%). ^{1}H NMR (400 MHz, $CDCl_3$): δ_H 9.90 (1H, s, CHO), 8.41 (2H, d, J = 9.2 Hz, ArHs), 7.43 (2H, d, J = 8.8 Hz, ArHs), 6.44 (1H, s, pyrrple-H), 2.32 (3H, s, CH_3), 2.03 (3H, s, CH_3).

2,5-Dimethyl-1-(3-nitrophenyl)-1*H*-pyrrole-3-carbaldehyde, 8f

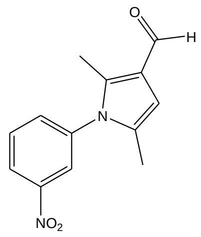

Yellow solid (61%). ^{1}H NMR (300 MHz, $CDCl_3$): δ_H 9.87 (1H, s, CHO), 8.39 – 8.35 (1H, m, ArH), 8.12 (1H, t, *J* = 1.8 Hz, 2.1 Hz, ArH), 7.78 (1H, t, *J* = 8.1 Hz, ArH), 7.61 – 7.58 (1H, m, ArH), 6.41 (1H, s, pyrrole-H), 2.30 (3H, s, CH_3), 2.01 (3H, s, CH_3).

2,5-Dimethyl-1-(4-(trifluoromethyl)phenyl)-1*H*-pyrrole-3-carbaldehyde, 8g

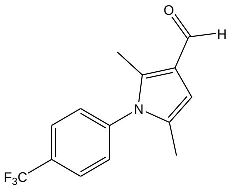

White solid (83%). ^{1}H NMR (600 MHz, $CDCl_3$): δ_H 9.88 (s, CHO), 7.81 (2H, d, *J* = 8.8 Hz, ArHs), 7.36 (2H, d, *J* = 8.8 Hz, ArHs), 6.41 (1H, s, pyrrole-H), 2.29 (3H, s, CH_3), 2.00 (3H, s, CH_3).

2,5-Dimethyl-1-(3-(trifluoromethyl)phenyl)-1*H*-pyrrole-3-carbaldehyde, 8h

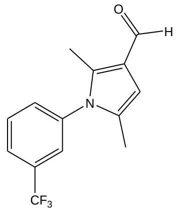

Light brown solid (87%). ^{1}H NMR (300 MHz, $CDCl_3$): δ_H 9.88 (1H, s, CHO), 7.77 (1H, d, *J* = 8.1 Hz, ArH), 7.69 (1H, t, *J* = 7.8 Hz, ArH), 7.50 (1H, s, ArH), 7.43 (1H, d, *J* = 7.8 Hz, ArH), 6.41 (1H, s, pyrrole-H), 2.29 (3H, s, CH_3), 2.00 (3H, s, CH_3).

2,5-Dimethyl-1-(2-(trifluoromethyl)phenyl)-1*H*-pyrrole-3-carbaldehyde, 8i

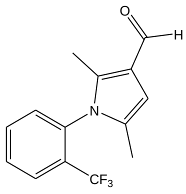

Brown solid (68%). ^{1}H NMR (300 MHz, $CDCl_3$): δ_H 9.87 (1H, s, CHO), 7.89 – 7.86 (1H, m, H), 7.77 – 7.64 (2H, m, ArHs), 7.29 – 7.26 (1H, m, pyrrole-H), 6.39 (1H, s, CH_3), 2.19 (3H, s, H_6), 1.90 (3H, s, CH_3).

1-(4-Methoxyphenyl)-2,5-dimethyl-1*H*-pyrrole-3-carbaldehyde, 8j

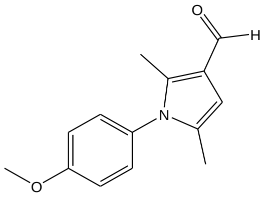

White solid (90%). ^{1}H NMR (400 MHz, $CDCl_3$): δ_H 9.86 (1H, s, CHO), 7.11 (2H, d, *J* = 8 Hz, ArHs), 7.01 (2H, d, *J* = 8.0 Hz, ArHs), 6.36 (1H, s, pyrrole-H), 3.87 (3H, s, OCH_3), 2.26 (3H, s, CH_3), 1.97 (3H, s, CH_3).

2,5-Dimethyl-1*H*-pyrrole-3-carbaldehyde, 8k

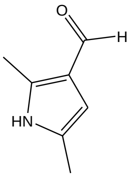

White solid (60 %). ^{1}H NMR (300 MHz, $CDCl_3$): δ_H 9.77 (1H, s, CHO), 8.75 (1H, s, N-H), 6.20 (1H, s, pyrrole-H), 2.49 (3H, s, CH_3), 2.21 (3H, s, CH_3).

1-Cyclohexyl-2,5-dimethyl-1*H*-pyrrole-3-carbaldehyde, 8l

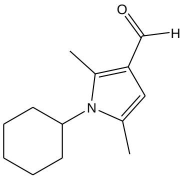

Light brown solid (85%). ^{1}H NMR (300 MHz, $CDCl_3$): δ_H 9.80 (1H, s, CHO), 6.26 (1H, s, pyrrole- H), 4.02 – 3.91 (1H, m, cyclic-H), 2.58 (3H, s, CH_3), 2.31 (3H, s, CH_3), 2.02 – 1.74 (7H, m, cyclic-H), 1.47 – 1.15 (3H, m, cyclic-H).

Table S1: Crystal data and structure refinement for compound **10e**.

**Formula**	$\mathbf{C_{22}H_{17}BrF_3NO_2}$
Formula Weight	464.27
Crystal System	orthorhombic
Space group	*Pbca*
a/(Å)	16.380(2)
b/(Å)	8.2052(11)
c/(Å)	29.790(4)
V/(Å^3)	4003.8(9)
Z	8
D(calc) [g/cm^3]	1.540
Mu(MoKa) [/mm]	2.098
F(000)	1872
Crystal Size [mm]	0.10 x 0.14 x 0.20
Temperature (K)	100 (2)
Dataset	-18: 21 ; -10: 10 ; -39: 36
Tot., Uniq. Data, R(int)	21981, 4967, 0.082
Observed Data [I > 2.0 sigma(I)]	2648
Nref, Npar	4967, 268
R, wR2, S	0.0751, 0.2017, 1.04
Max. and Av. Shift/Error	0.00, 0.00
Min. and Max. Resd. Dens. [e/Å^3]	-1.63, 1.71
Radiation (Å)	MoKa 0.71073

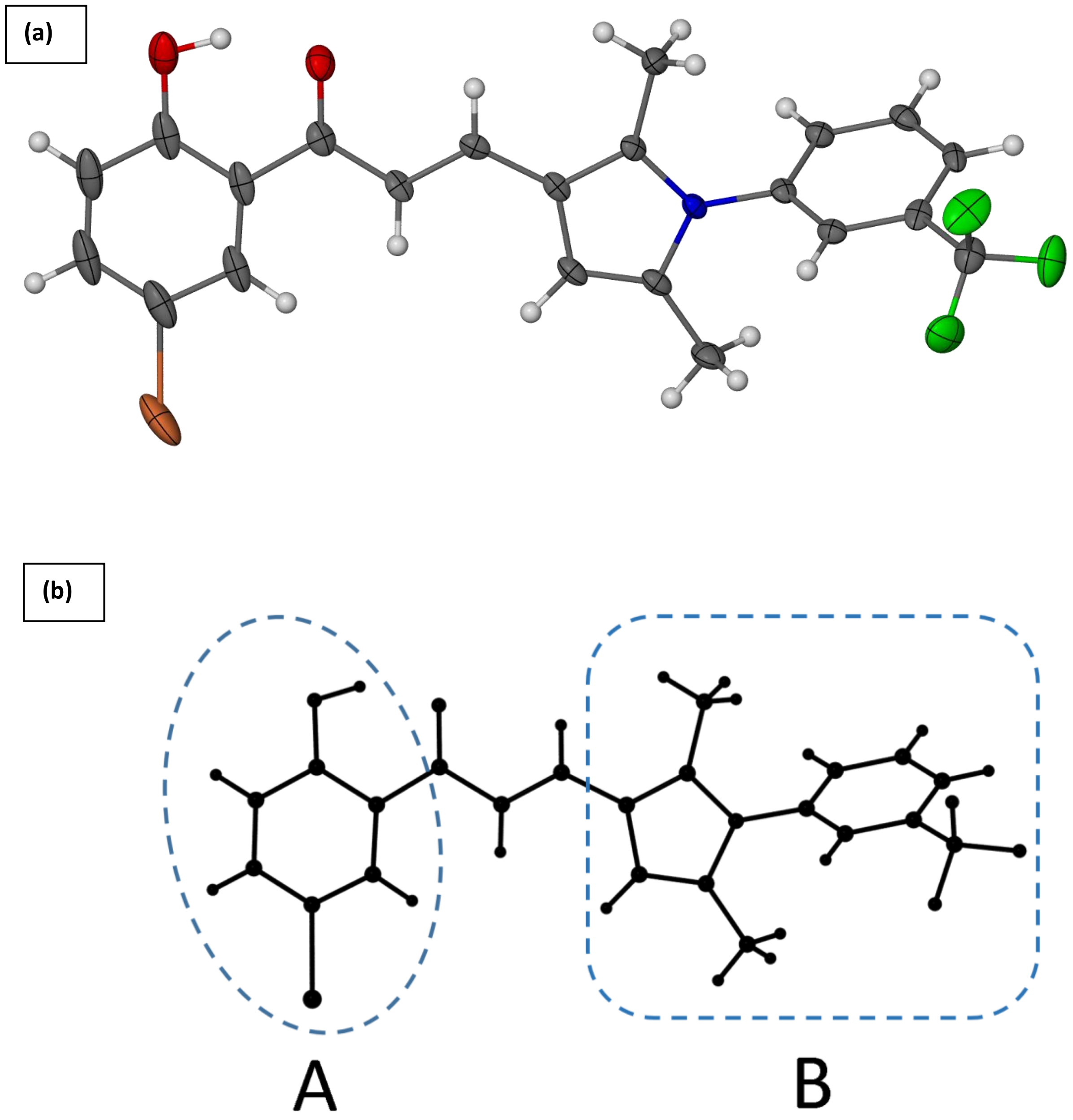

Figure S1: (a) Molecular structure of **10e** with all non-hydrogen atoms refined anisotropically and the ellipsoids are drawn at the 50% probability level. **(b)** Structure of **10e** showing a *trans*-configuration about C(8A)-C(9A) bond.

τ		τ/(°)
C13A-N1A-C16A-C21A	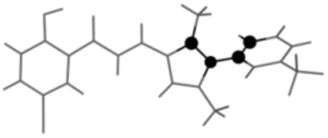	-66.9(5)
C12A-N1A-C16A-C21A	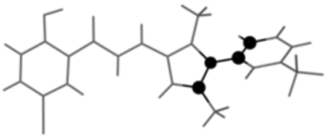	111.8(4)
C12A-N1A-C16A-C17A	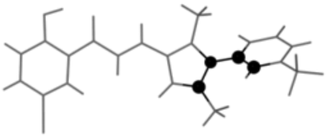	-69.2(5)
C13A-N1A-C16A-C17A	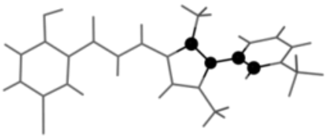	112.2(4)

Figure S2: Illustration of torsion angles around the N(1A)-C(16A) bond of **10e**. The torsion angles with values displayed alongside the angles.

Table S2: Residual percentage viability of cytotoxicity (HeLa cell viability) data for solutions of prepared compounds

Comp	HeLa %Viability	SD	Comp	HeLa %Viability	SD
**10a**	>100	1.6	**10l**	99.2	2.5
**10b**	>100	0.2	**11a**	78.1	10.8
**10c**	>100	4.5	**11b**	99.3	6.9
**10d**	>100	0.47	**12**	84.7	13.4
**10e**	>100	8.8	**13**	>100	11.0
**10f**	>100	1.6	**14**	99.4	3.2
**10g**	>100	5.3	**15**	98.9	4.2
**10h**	>100	0.46	**16**	>100	8.6
**10i**	51.2	10.6	**17**	>100	1.9
**10j**	>100	9.7	**18**	79.9	2.1
**10k**	>100	4.4	**19**	81.2	3.0
**EMT**	0.76 μM		**-**	-	

Appendices: Selected NMR spectra of some compounds synthesised

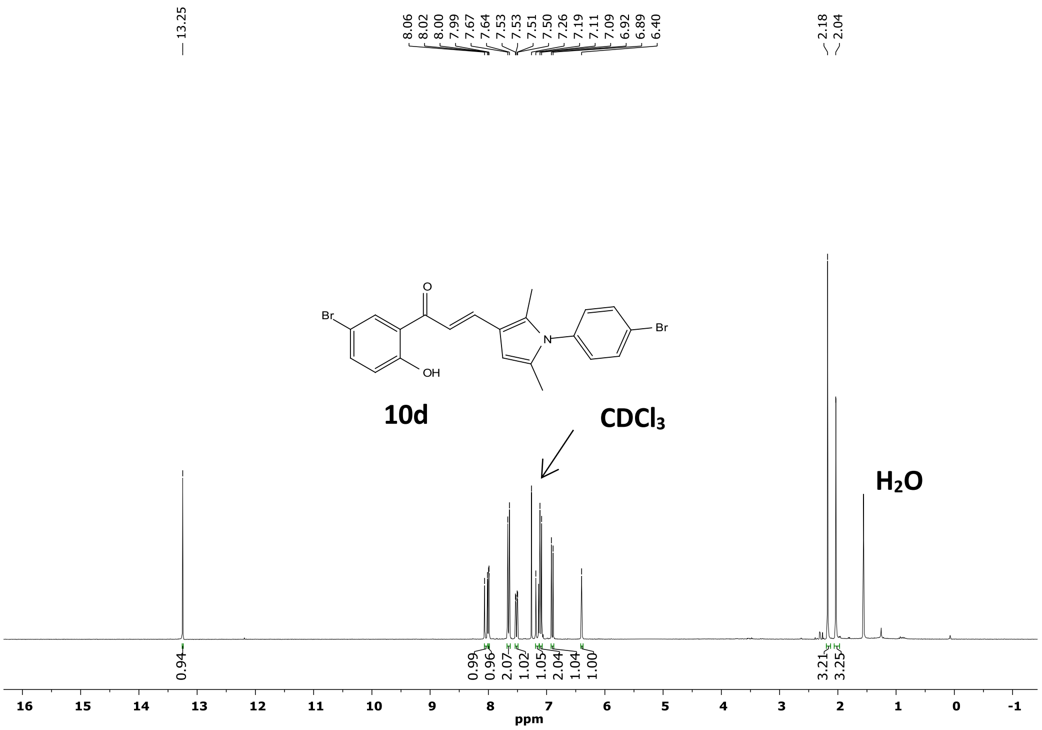

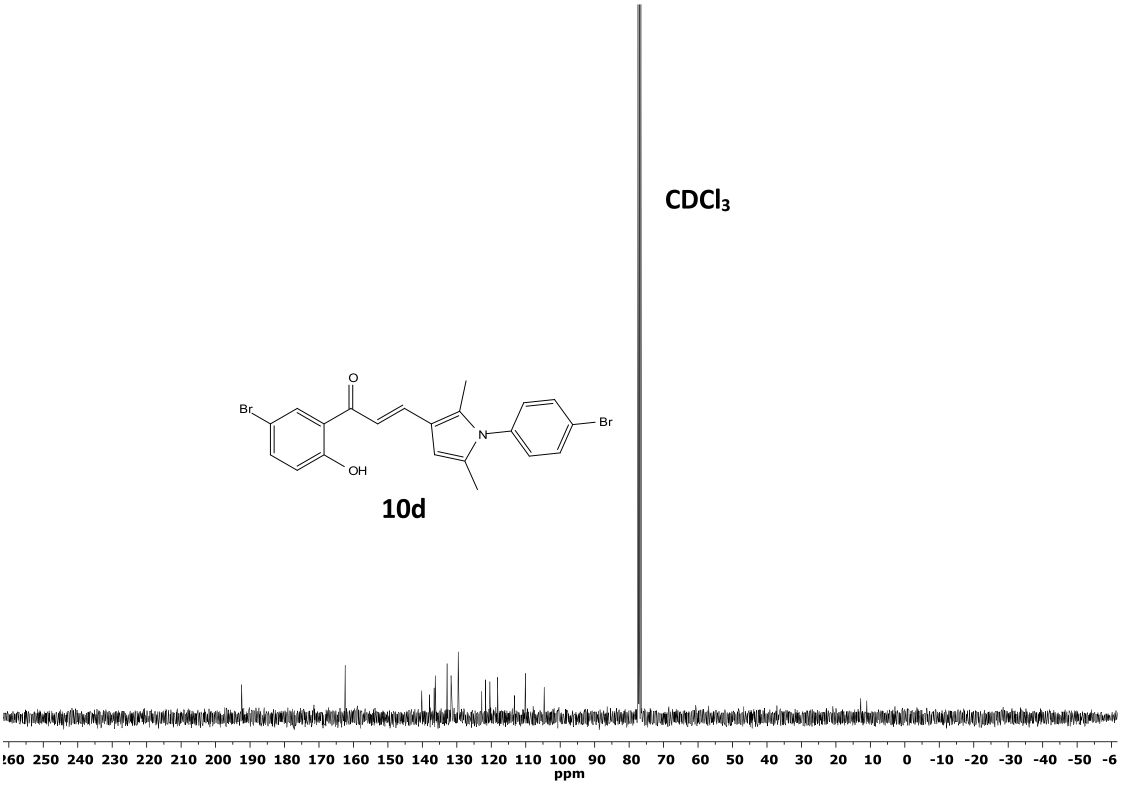

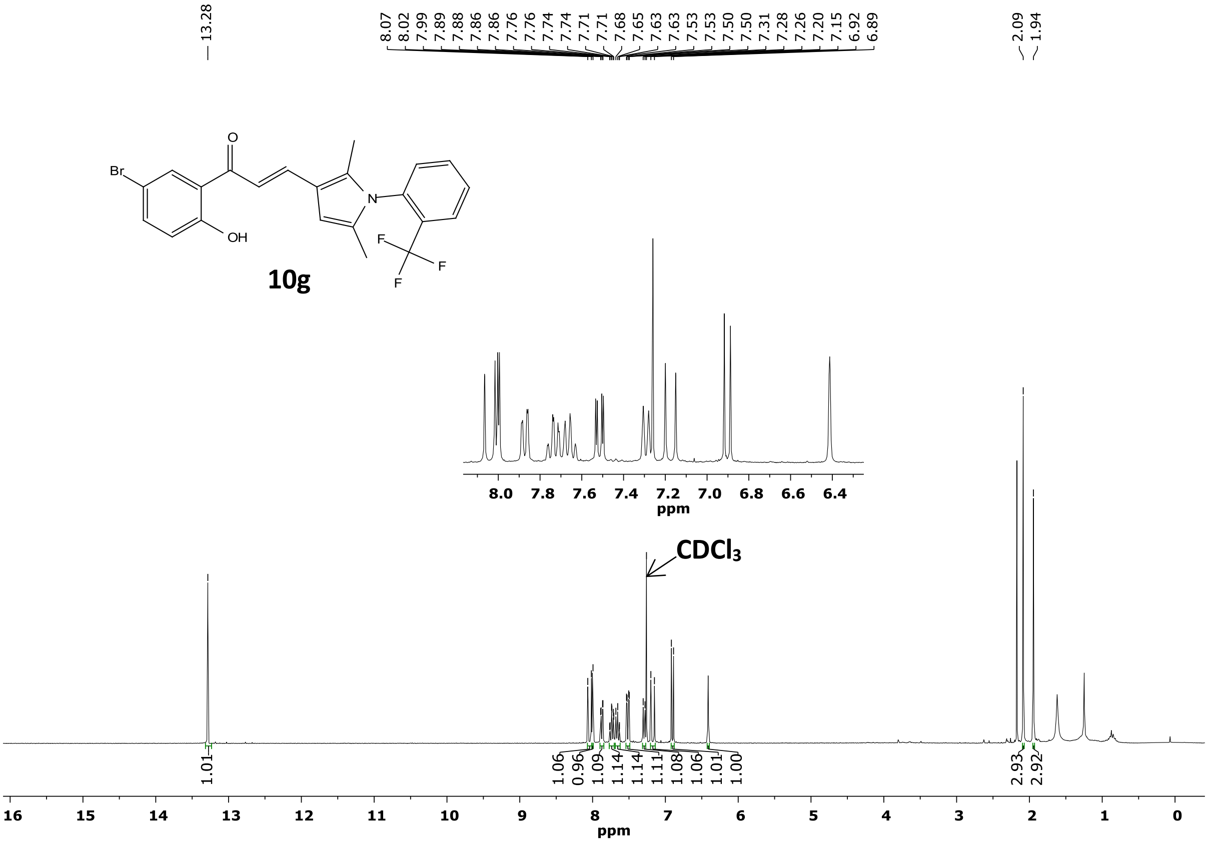
13.28
8.07 8.02 7.99 7.89 7.88 7.86 7.76 7.76 7.74 7.74 7.71 7.71 7.68 7.65 7.63 7.63 7.53 7.53 7.50 7.50 7.31 7.28 7.26 7.20 7.15 6.92 6.89
2.09 1.94
Br
O
OH
N
F
F
F
10g
8.0 7.8 7.6 7.4 7.2 7.0 6.8 6.6 6.4
ppm
CDCl3
1.01
1.06 0.96 1.09 1.14 1.14 1.11 1.08 1.06 1.01 1.00
2.93 2.92
16 15 14 13 12 11 10 9 8 7 6 5 4 3 2 1 0
ppm

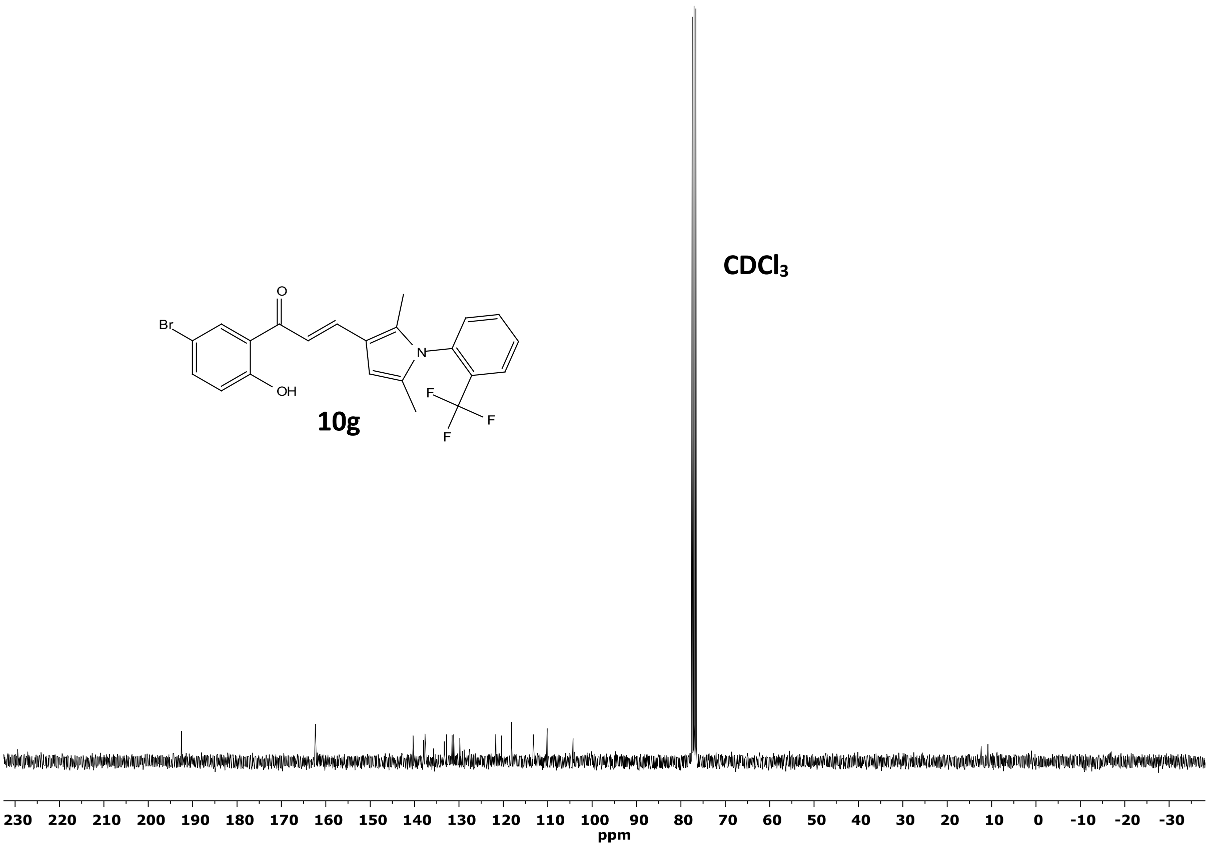
CDCl3
Br
O
OH
N
F
F
F
10g
230 220 210 200 190 180 170 160 150 140 130 120 110 100 90 80 70 60 50 40 30 20 10 0 -10 -20 -30
ppm

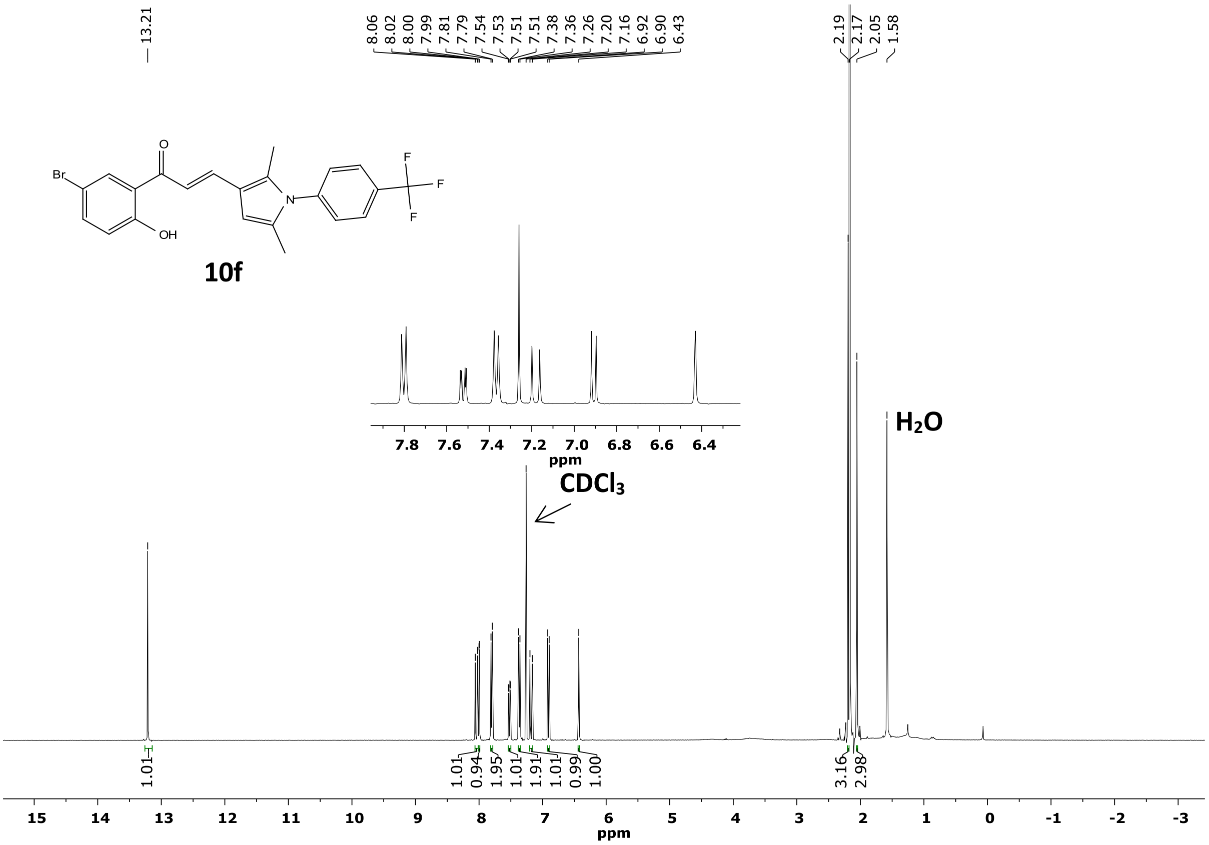
13.21
8.06 8.02 8.00 7.99 7.81 7.79 7.54 7.53 7.51 7.51 7.38 7.36 7.26 7.20 7.16 6.92 6.90 6.43
2.19 2.17 2.05 1.58
Br
O
OH
N
F
10f
H_2O
$CDCl_3$
1.01 1.01 0.94 1.95 1.01 1.91 1.01 0.99 1.00 3.16 2.98
ppm

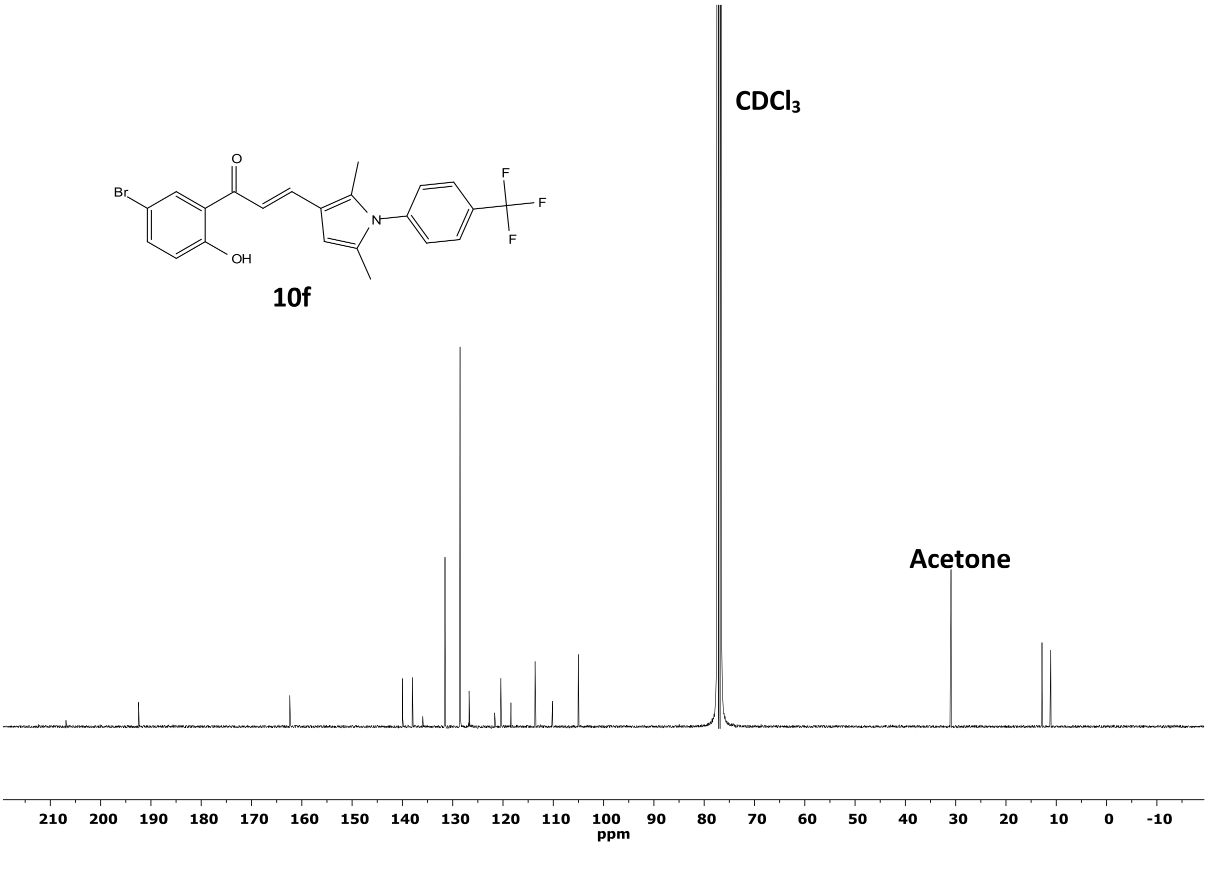
$CDCl_3$
Br
O
OH
N
F
10f
Acetone
ppm

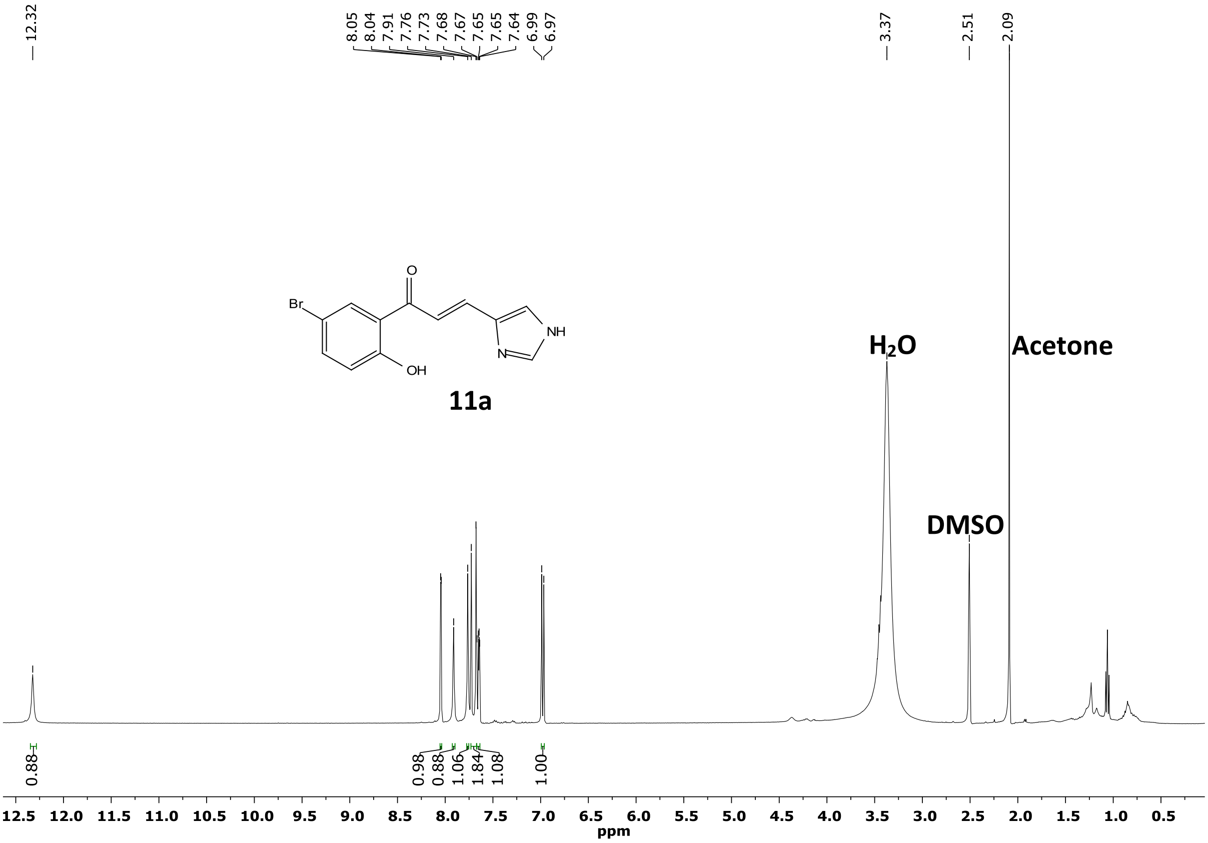

12.32
8.05
8.04
7.91
7.76
7.73
7.68
7.67
7.65
7.65
7.64
6.99
6.97
3.37
2.51
2.09
Br
O
NH
N
OH
11a
H_2O
Acetone
DMSO
0.88
0.98
0.88
1.06
1.84
1.08
1.00
12.5 12.0 11.5 11.0 10.5 10.0 9.5 9.0 8.5 8.0 7.5 7.0 6.5 6.0 5.5 5.0 4.5 4.0 3.5 3.0 2.5 2.0 1.5 1.0 0.5
ppm

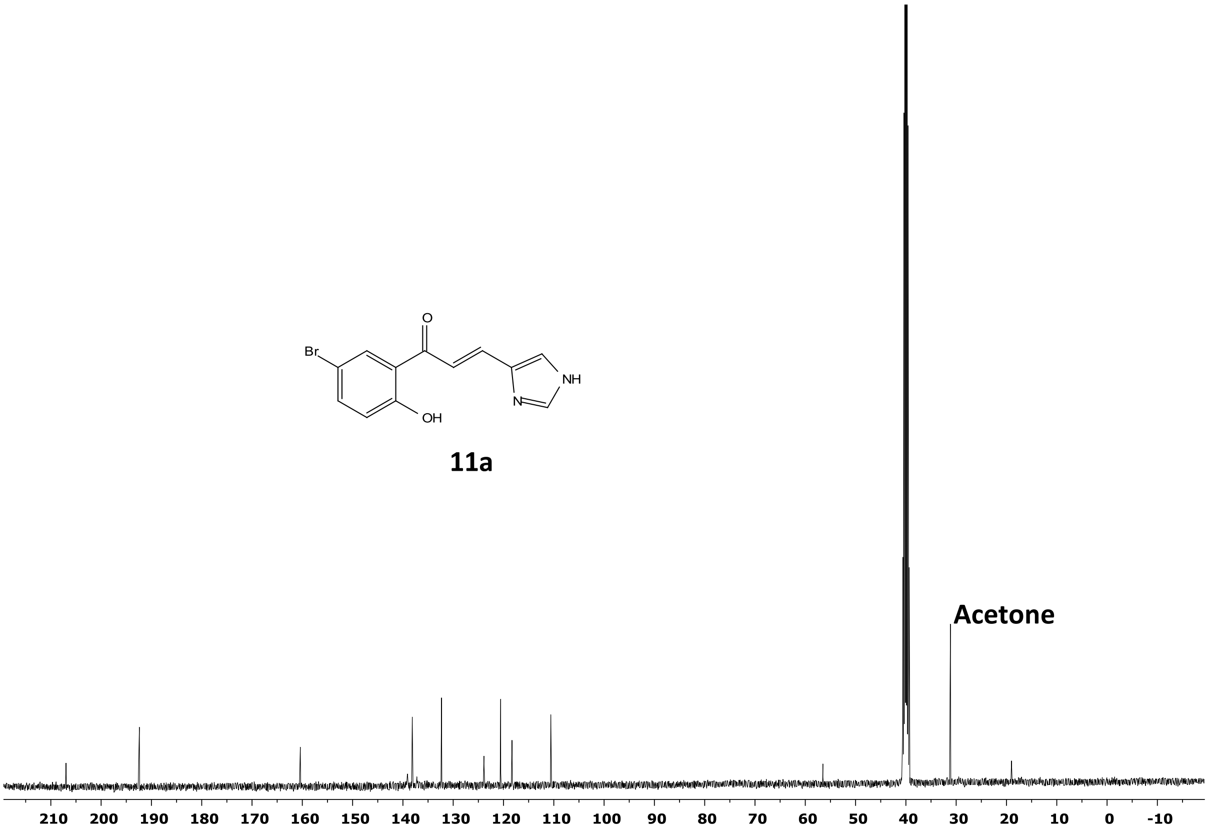

Br
O
NH
N
OH
11a
Acetone
210 200 190 180 170 160 150 140 130 120 110 100 90 80 70 60 50 40 30 20 10 0 -10
ppm
